# Characterization of isolates used in bacterial-based strategies for accelerated carbonation of lime mortars

**DOI:** 10.1128/aem.00683-25

**Published:** 2025-09-23

**Authors:** Franco Grosso Giordano, Quinten Mariën, Nele De Belie, Carlos Rodriguez-Navarro, Nico Boon

**Affiliations:** 1 Center for Microbial Ecology and Technology (CMET), Ghent University 26656, Ghent, Belgium; 2 Magnel-Vandepitte Laboratory, Department of Structural Engineering and Building Materials, Ghent University 26656, Ghent, Belgium; 3 Department of Mineralogy and Petrology, University of Granada 16741, Granada, Spain; Washington University in St. Louis, St. Louis, Missouri, USA

**Keywords:** carbonation, lime-based, mortar, biomineralization, alkaliphilic bacteria

## Abstract

Portland cement largely replaced hydraulic lime over the past century because of its rapid hardening. Achieving earlier hardening in lime through faster carbonation is thus essential to help overcome one of lime’s limiting qualities. Here, we isolated two alkaliphilic bacteria, *Shouchella clausii* and *Shouchella patagoniensis,* from a lime mortar wall. *S. clausii* was then further grown in high pH (>11) by adaptive laboratory evolution to acclimate a third isolate. Bacterial suspensions of all three isolates were followed for 14 days in serum bottles at pH 11, and gas composition of the headspace, intact/damaged cell populations, and pH were measured. In parallel, lime mortar samples were incubated in a closed environment with bacterial suspension of the isolates and analyzed with thermogravimetric analysis after 7 and 14 days to quantify carbonation. *S. patagoniensis* produced more CO_2_, close to the estimated maximum CO_2_ uptake rate of lime, and carbonated the lime mortars to a larger extent than the other isolates. Finally, the bacterial suspensions were directly mixed with lime. A linear and homogeneous carbonation of the paste samples was measured compared to water-based pastes, and the development of Liesegang patterns was observed upon phenolphthalein spreading. All this indicated that the organic addition altered the carbonation dynamics of the material, although bacteria did not accelerate carbonation relative to media alone and inhibited it relative to water-based paste. Still, a relationship between bacterial activity, CO_2_ emission, and carbonation rate was established, but practical aspects of bacterial delivery into lime must be addressed.

**IMPORTANCE:**

Portland cement is the dominant binder used in most construction today, but until last century, lime was the ubiquitous construction material. The increase in use of cement has sprung from its higher strength and faster hardening; yet, lime still remains a relevant material, particularly in masonry structures and the built heritage. As such, novel lime materials are necessary to tackle some of the current limitations of lime, such as earlier hardening, which would not only make lime easier to work with but would also limit failure due to environmental conditions. As existing strategies to speed up lime hardening have had limited uptake due to their reliance on expensive and often toxic chemicals, the need for novel solutions is in place. We show that bacterial-based strategies could be a viable option to go beyond the limitations of current strategies, but limitations are in place.

## INTRODUCTION

The last century saw a rise in the use of Portland cement as the dominant construction material, and with it, its use in built heritage restoration (1). Yet, the use of cement for the conservation and repair of these historical structures poses a huge risk as the material is not compatible with the masonry structures. For example, due to lime mortar’s higher water content, lower binder/aggregate ratio (by mass), and lack of hydration reactions, lime mortars have larger pores and higher porosity than cement. This higher porosity allows for better conduction of humidity, resulting in lower moisture or salt damage to masonry (2). Lime, a material used for millennia in masonry, is therefore more appropriate, yet it has some drawbacks. One of the main reasons for the increased adoption of cement instead of lime was due to the slow hardening process of lime (1).

The process of setting and hardening of aerial lime mortars is through the reaction of hydrated lime with CO_2_ (equations 1-3):


(1) $$CO_{2}\left(aq\right)+\;H_{2}O↔\;H_{2}CO_{3}\;↔HCO_{3}^{-}+H^{+}↔CO_{3}^{2-}+2H^{+}$$

(2) $$\;Ca{\left(OH\right)}_{2}↔Ca^{2+}\left(aq\right)+2OH^{-}$$

(3) $$Ca^{2+}\left(aq\right)+CO_{3}^{2-}\left(aq\right)↔CaCO_{3}$$

The main rate-limiting step in the overall carbonation of hydrated lime mortars is the diffusivity of CO_2_ to the pore system where the dissolution of CO_2_ will occur (equation 1) (3). In atmospheric conditions, this reaction can be very slow with reports of 1,700-year-old mortars that are still not fully carbonated (4). CO_2_ diffusivity can be affected by the pore size distribution of the matrix and the water content. Small pore size leads to lower gas exchange, whereas a high water saturation directly inhibits the diffusion of CO_2_ as its diffusion rate can be 10,000 times lower than in air (5). Moreover, as carbonation occurs from outside to inside the material, the deepest sections of the matrix take longer to carbonate (3). Under ideal conditions, i.e., a thin layer of lime putty paste exposed to high CO_2_ concentrations as tested by Van Balen (5), CO_2_ uptake per unit time reached an average maximum ranging from 3.99 × 10^−4^ to 2.84 × 10^−3^ mmol of CO_2_ absorbed per millimole of lime per second (1/s). It follows that one strategy to overcome the slow hardening of lime-based mortars would involve speeding up carbonation to such rates. Hence, shifting the equilibrium of equation 1 to the right is essential to speed up carbonation at an early age.

Three parameters can be influenced to improve carbonation rates: increasing the temperature, limiting humidity to an optimal range of 40 to 80% (6), and increasing CO_2_ concentration ([CO_2_]) up to approximately 20% (7). Of all three, the latter is the most studied, and strategies have been engineered to use in lime materials. It can be affected through increasing diffusivity of CO_2_, by increasing locally the partial pressure of CO_2_ (*p*CO_2_) and/or CO_2_ concentration in air, or increasing the dissolution of CO_2_ in water to produce bicarbonate ions (equation 1). Increasing CO_2_ diffusivity can be achieved by changing the microstructure of the material to allow faster airflow (i.e., by increasing the porosity, pore size, and pore connectivity). Nevertheless, this can lead to detrimental changes in mechanical properties, and the outside-to-inside process of carbonation remains a hurdle. Changes in *p*CO_2_ or CO_2_ concentration, usually achieved in controlled environments, are effective up to a maximum, ranging from 5% (8) to 20% [CO_2_] (7), after which carbonation can lead to the formation of an impervious layer of CaCO_3_ that completely inhibits the progress of carbonation (5). Also, changes in *p*CO_2_ are heavily impacted by humidity (9), as the dissolution/hydration of CO_2_ (equation 1) is the governing rate-limiting step of lime carbonation. Lastly, too high a concentration of CO_2_ can lead to a high heat release from the exothermic reaction of carbonation, leading to premature drying and the stopping of carbonation (10). All this highlights that optimal solutions for carbonation need to strike a balance between the increase in CO_2_ availability through the entire matrix while maximizing the dissolution of CO_2_.

Multiple strategies for enhancing carbonation already exist. Of the many strategies implemented (3), the most positive results in terms of accelerating carbonation have been observed with some additives, especially organic ones, such as vegetable matter (11), plant extracts (12), and animal products (13). A little-explored strategy is, however, the use of microbial-mediated carbonation. In principle, microorganisms possess multiple qualities that make them attractive as early hardening additives. Firstly, there is the use of microbially induced carbonate precipitation (MICP). In MICP, metabolites from microbial activity—of which those primarily studied have been bacterial—generally lead to the formation of CO_3_^2-^ ions which can then react with free Ca^2+^ to form CaCO_3_ (equation 3) (14). One of these pathways is the release of CO_2_ through the breakdown of organic acids during respiration. This can be used to increase the rate of carbonation with the possibility of creating a CO_2_ source working from the inside of the material. Lopez-Arce et al. showed that such a strategy was also possible using a yeast fermentation system—another microbial source of CO_2_ different to bacteria—to carbonate Ca(OH)_2_ nanoparticles applied for the consolidation of limestone samples placed in a separate container (15). Moreover, a patent application exploring microbially mediated carbonation saw positive results when directly mixing a bacterial suspension with the mortar, achieving at least 1.2 times higher compressive strengths than reference mortars (16). Still, here, the addition of urea or organic acids was the biggest enhancer of the compressive strength, while bacteria activity led to a small cumulative increase. Secondly, another benefit is that the CO_2_ release by microorganisms is not instantaneous, preventing the immediate precipitation of CaCO_3_. This is a problem in CO_2_ sources derived from chemical compounds, as it can be detrimental to the fresh properties (rheology) of lime materials. For example, acceleration of carbonation can be achieved using carbamates which in contact with water decompose, releasing CO_2_ right upon mixing with the fresh lime paste (17). The study of carbamates has nonetheless recently been revived and could be promising in the future (18). Thirdly, the diversity of microbes, in particular bacterial species, offers a large array of options to optimize microbially mediated carbonation, through the choice of metabolic pathways and selection of microbial growth kinetics (14). Finally, bacteria also release extracellular polymeric substances (EPS), composed of a mixture of proteins, polysaccharides, and other organic compounds. EPS have been observed to control the morphology and kinetics of the biomineralization of CaCO_3_, and this has further effects on the structure-function of calcite (19). Once again, this further suggests that microbial-mediated carbonation of lime is a sensible strategy.

It is worth noting that bacterial additives have been previously engineered for construction materials. For example, work on bio-additives in cement mixtures is very well-established for bioconsolidation or as self-healing agents (20). Similar hurdles are faced in terms of application in fresh cement as in fresh lime, particularly in terms of incorporation of the bacterial additives to the cement mixtures. The cement environment can be very harsh due to pH reaching >12 in a fresh state, as well as the small pore size of the cement matrix. For this reason, protective mechanisms for the bacteria are often implemented, which improves survivability in the long term, while alkaliphiles are more often selected to better survive in the high pH environment (21). MICP has also been used for consolidation of limestone to restore and improve lost mechanical properties due to weathering through the creation of new calcium carbonate cement by applying bacteria (22, 23) or through the activation of the indigenous carbonatogenic bacteria already present in the stone substrate (24). Nevertheless, the conditions of application in these works have been very different from those expected in fresh lime mortars, specifically due to the close-to-neutral pH of limestone and the superficial application of bacteria.

As such, inspired by the engineered process of MICP used in cement and concrete, here, we study the potential of bacterial-based strategies for early age hardening of hydrated lime. The approach taken seeks to increase the availability of CO_2_ through the bacterial metabolism of organics, which should speed up equation 1, leading to a faster carbonation in the presence of bacteria than without them. This work serves as a proof of concept for such a strategy. Importantly, the focus is doing so at an early age (up to 14 days), which would produce faster setting lime mortars, overcoming the main limiting quality for the wider use of hydrated lime mortars (3). Therefore, we performed an isolation campaign from which three isolates were selected and studied in-depth for their alkalophilicity, their CO_2_ production rates, their capacity to carbonate lime mortars, and, lastly, their effect on hydrated lime when directly incorporated into the material.

## MATERIALS AND METHODS

### Environmental isolation campaign

**Fig 1:**
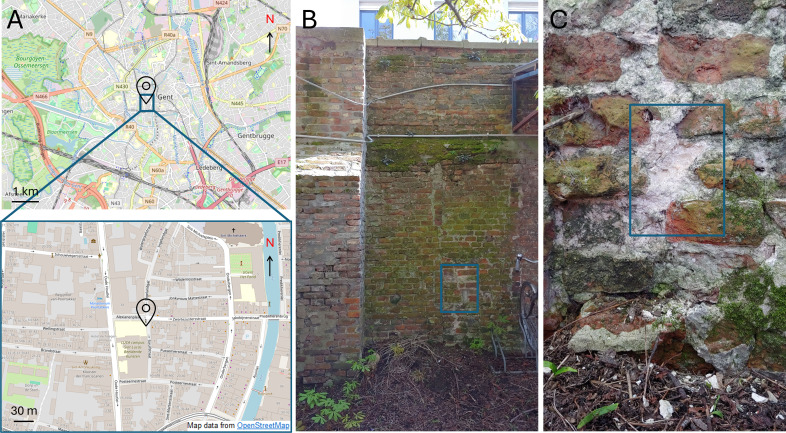
(**A**) Top: location of sampling of lime mortar masonry wall in Gent, Belgium. Bottom: zoom-in of location on the eastern wall. Map data obtained from OpenStreetMap.org. (**B**) Front view of the sampling area (blue square) on the northeast wall, where the least amount of biological degradation was observed. (**C**) Zoom-in of the cleaned wall prior to sampling the internal lime mortar (blue square).

A lime mortar from a wall in Ghent, Belgium, was sampled (Fig. 1). A part of the mortar sample was ground and analyzed under X-ray diffraction (XRD) to disclose its mineralogy with a Siemens D5000 (Munich, Germany) (measurement parameters: Cu-Kα radiation, 40 kV, 40 mA, 5 to 90 °2θ exploration range). After analysis, the mortar was placed in a 37% HCl solution and stirred for 30 min. The suspension was filtered, and the acid-insoluble residue was collected and washed thrice. XRD was performed again on the residue. The XRD analysis confirmed the mortar was indeed a lime mortar, composed of calcite (as binder) and quartz (as aggregate) (Fig. S1).

The mortar was then placed under UV light for 10 min on all sides to sterilize the surface. Using a surface-sterilized mortar and pestle, the lime mortar was ground, and media were inoculated with mortar. A neutral (pH 7.0–7.4) low nutrient medium, R2A (yeast extract 0.5 g/L; proteose peptone 0.5 g/L; casein hydrolysate 0.5 g/L; glucose 0.5 g/L; soluble starch 0.5 g/L; Na-pyruvate 0.3 g/L; K_2_HPO_4_ 0.3 g/L; MgSO_4_ anhydrous 0.024 g/L), and two high pH media, Horikoshi (glucose 10.0 g/L, polypeptone 5.0 g/L, yeast extract 5.0 g/L, K_2_HPO_4_ 1.0 g/L, MgSO_4_ × 7 H_2_O 0.2 g/L, 10 g/L Na_2_CO_3_; final pH 10) and R2A adjusted to pH 9.2 with carbonate buffer, were used. The enrichment was grown for 48 h and then diluted 10^−3^ to 10^−5^ and plated on agar plates of the same media. Morphologically distinct colonies were transferred to a new plate and streak cleaned three times to ensure purity of the isolate. All samples were incubated at 28°C between 24 h and 120 h.

### Microbe maintenance

All isolates were grown and maintained in tryptic soy broth (TSB: pancreatic digest of casein 17.0 g/L, peptic digest of soybean 3.0 g/L, glucose 2.5 g/L, NaCl 5.0 g/L, di-potassium hydrogen phosphate 2.5 g/L) or tryptic soy agar (TSA: pancreatic digest of casein 17.0 g/L, peptic digest of soybean 3.0 g/L, NaCl 5.0 g/L, agar 14 g/L). For each experiment, a double transfer was performed to reduce carryover effects from agar plates or stationary phases. All growth conditions were at 28°C and 140 RPM unless stated otherwise.

### Isolate selection

Matrix-assisted laser desorption-ionization time-of-flight mass spectrometry (MALDI-TOF MS), as performed in reference 25, was used to dereplicate the isolates through the comparison of spectral differences. 16S rRNA gene sequencing was performed on the remaining reference strains. With the species-level identification, isolates were excluded based on their classification as biosafety level 2, i.e., microbes that pose moderate hazards to humans and the environment, according to the BacDive database (26).

Then, isolates were selected for their growth capacity at high pH. Isolates were inoculated to OD_600_ 0.01 in microtiter plates in TSB adjusted to pH 10 using carbonate buffer (TSB pH 10: pancreatic digest of casein 17.0 g/L, peptic digest of soybean 3.0 g/L, glucose 2.5 g/L, NaCl 5.0 g/L, NaHCO_3_ 3.9 g/L, Na_2_CO_3_ 5.7 g/L). The best-growing isolates were then grown in TSB adjusted to pH 11 using 3-(cyclohexylamino)-1-propanesulfonic acid (CAPS) buffer (TSB pH 11: pancreatic digest of casein 17.0 g/L, peptic digest of soybean 3.0 g/L, glucose 2.5 g/L, NaCl 5.0 g/L, CAPS 22.13 g/L) (TSB pH 11).

After reducing the number of isolates based on their alkalophilicity, the CO_2_ production rate of the best-growing bacteria was measured in a serum bottle experiment in TSB. A 1:100 inoculation was made in fresh TSB, and the isolates were grown for 24 h to achieve full growth. The TSB suspension was centrifuged at 1,500 relative centrifugal force (RCF) for 10 min and resuspended in fresh TSB. Serum bottles of 30 mL volume with 10 mL of the bacterial suspension were prepared, capped with butyl rubber stoppers, and sealed. The samples were incubated under constant shaking. A total of 1.5 mL of the headspace of the bottles was sampled using a syringe and needle at 0, 7, and 14 days, and CO_2_ levels were measured using gas chromatography (see below).

The selected isolates were then deposited at the Belgian Coordinated Collections of Microorganisms (BCCM), and the 16s rRNA gene sequences were submitted to the Database Resources of the National Center for Biotechnology Information (27). The isolates tested in this work are *Shouchella clausii* (LMG 33235, GenBank accession no. PV163962), *Shouchella patagoniensis* (LMG 33400, GenBank accession no. PV163981), and *Shouchella clausii* t10 (LMG 33236, GenBank accession no. PV163963).

### 16S rRNA gene sequencing

Clean-streaked colonies were picked with a pipette tip and resuspended in 20 µL PCR water and incubated at 98°C for 10 min to prepare the PCR sample. PCR was performed using Phusion Plus Green PCR Master Mix (Thermo Fisher Scientific) in 20 µL reactions containing 10 µL 2× Phusion Plus Green PCR Master Mix, 1 µL of 10 µM primer 27f AGAGTTTGATCMTGGCTCAG, 1 µL of 10 µM primer 1492r TACGGYTACCTTGTTACGACTT, 7 µL PCR water, and 1 µL sample. Amplification was run including initial denaturation for 30 s at 98°C, followed by 30 cycles of 10 s denaturation at 98°C, 10 s anneal at 55°C, and 1 min extension at 72°C. A final elongation step was included at 72°C for 5 min.

The product was run for 30 min at 100 V on a 2% agarose gel and was visualized using HDGreen Plus Dye (Isogen) according to the manufacturer’s instructions. Positive PCR fragments were purified using the GeneJET PCR Purification Kit (Thermo) and eluted in elution buffer. A total of 4 µL from a 5 µM stock of one of the primers was added to 10 µL purified PCR product, for both forward and reverse sequencing reactions. The reactions were sent out to LGC Genomics (LGC Genomics GmbH, Berlin, Germany). The resulting sequences were blasted using NCBI’s BLAST.

### Adaptive laboratory evolution for high pH tolerance

Adaptive laboratory evolution of an isolate was performed on a microtiter plate. The selected isolate was inoculated to OD_600_ 0.01 in microtiter plates containing 200 µL TSB pH 11 in triplicate. After growth, the best-growing replicate was reinoculated with a 1% inoculum in 200 µL TSB pH 11. The experiment was carried out for 10 consecutive transfers. The final grown isolate was deposited at the BCCM.

#### Generation calculation based on OD_600_ measurements

To determine the number of generations (“*n*”) in a bacterial culture based on OD_600_ measurements, we utilized the following equation:


(4) $$n=\frac{\log\left(OD_{final}\right)-log\left(OD_{initial}\right)}{\log2}$$

where OD_initial_ is the optical density at the beginning of the measurement period, OD_final_ is the optical density at the end of the measurement period, and log2 accounts for the doubling nature of bacterial growth.

#### Models and data analysis

All selected isolates were grown in TSB pH 11 in nine replicates each. The OD/time data were log-transformed, and a Gompertz model was used for curve fitting for each growth curve. Outliers were removed based on Iglewicz and Hoaglin’s robust test for multiple outliers. The growth rate and the relative standard error (1σ) for each isolate were estimated from the model for each isolate.

#### Statistical analysis on growth models

A two-tail, two-sample equal variance, *t*-test (α = 0.05) was performed to examine the effect of adaptive laboratory evolution (ALE) on the final grown isolate’s maximum growth rate, OD, and lag phase. All tests were performed in R.

### Microbial activity measurements and carbonation of lime mortar samples

The microbial activity of the selected isolates was followed in a closed, glass serum bottle experiment (Fig. S2A). In parallel, and using the same bacterial suspension, the carbonation rate of lime mortar samples incubated in a close environment was followed (Fig. S2B). For the bacterial suspension, a 1:10 inoculation was made in fresh TSB pH 11. The culture was centrifuged at 1,500 *g* for 10 min, and the pellet was resuspended in fresh TSB pH 11.

#### Microbial activity in a high pH environment

Four technical replicates were prepared in serum bottles with 10 mL of the bacterial suspensions resuspended in fresh medium, capped with butyl rubber stoppers and sealed. The samples were incubated and shaken. Sampling of 250 µL of bacterial suspension was performed from the serum bottle at 0, 1, 7, 10, and 14 days for pH measurement and flow cytometry analysis. A total of 2 mL from the gas phase in the headspace of the bottles was sampled at 0, 1, 3, 5, 7, 10, and 14 days to measure O_2_ and CO_2_ levels. The headspace pressure was also measured with a tensiometer (GMH 3111 equipped with a 603310 MSD 2.5 BAE sensor, Greisinger) and needle. To test the aerobic requirements of the bacteria, two serum bottles were briefly reopened at day 7 of the experiment using a needle and filter, to ensure sterility, and the internal atmosphere of the bottle was stabilized with the external atmosphere. Serum bottles with only water and TSB pH 11 were prepared and handled equally as controls. One serum bottle also contained TSB + NaN_3_. NaN_3_ was used to inhibit bacterial growth for the experimental section on the carbonation of lime mortar samples (see below). Such control was also prepared here to follow the effect of NaN_3_ on the potential release of CO_2_ from the uninoculated media in the control lime mortar samples experiment. No effect was observed though (Fig. S3).

#### Carbonation of lime mortar samples

A lime mortar mixture was prepared using a 1:3 lime:aggregate ratio (by volume) with a water:binder ratio of 0.85 (by volume). The aggregate used was a standard normalized quartz sand that fulfills the grain size distributions based on EN 196-1 (>98% SiO_2_). The hydrated lime was a CL 90-S (Lhoist, Belgium). The mortar was cast into a silicone mold containing wells of 1.4 × 1.4 × 1 cm^3^ (w × l × h). A layer of gauze and two layers of filter paper were placed on top, and 1.5 kg of weight was applied to the mortar, adapting the standard mortar casting technique to smaller samples. They were left to rest for 1 h and then placed at 100°C for 1.5 h for further drying.

After drying, two lime mortar samples were placed inside a Falcon tube separated by a layer of sterile aluminum paper from 9 g of sterilized cotton (Fig. S2B). Using a pipette, 10 mL of the bacterial suspensions resuspended in fresh medium was added to the cotton and then the tube was closed firmly. Tubes with cotton soaked with water (referred to as Water), tubes with cotton soaked with TSB pH 11 and 0.02 % NaN_3_ (referred to as TSB), and tubes with a dry cotton (referred to as Air) were prepared as controls. At 7 and 14 days, the soaked cotton was removed from the tube and the area was cleaned with a fresh cotton and ethanol, without coming into contact with the lime mortar samples. The soaked cotton was then replaced with clean, dry cotton, and the lime mortar samples were further stored in the closed tubes. Duplicate tubes for each treatment were prepared and tested at each time point (7 and 14 days of incubation), yielding a total of four tubes per treatment. All the tubes were stored inside a zip-lock bag. Soda lime with a CO_2_ indicator (Merch, Darmstadt, Germany) was placed in each tube to ensure proper storage and to halt any further mortar sample carbonation until thermogravimetric analysis (TGA).

#### Flow cytometry

Total cell concentrations and intact/damaged populations were measured as per reference 28. Briefly, an Attune NxT flow cytometer was used (Thermo Fisher Scientific, Belgium) with BRxx configuration, equipped with a blue (488 nm, 50 mW) and red laser (638 nm, 50 mW), two fluorescence detectors with bandpass filters (BL1: 530/30 nm, BL3: 695/40 nm), and two scatter detectors on the 488 nm laser (FSC: 488/10 nm, SSC: 488/10 nm). The flow cytometer was operated with Attune focusing fluid (Thermo Fisher Scientific, Belgium) as sheath fluid. Samples were diluted between 10^−1^ and 10^−3^ prior to staining for intact/damaged population identification with a combination of SYBR Green I (SG, 100× concentrate in 0.22 µm-filtered dimethyl sulfoxide [DMSO], Invitrogen, Belgium) for intact cells and propidium iodide (20 mM in DMSO, Invitrogen, Carlsbad, CA) for damaged cells (SGPI). The samples were incubated for 20 min at 37°C in the dark. Samples were then analyzed in triplicate in either fixed volume mode or maximum event count (20,000 events) at a flow rate of 100 µL/min.

#### Gas chromatography

The gas-phase composition of the headspace was analyzed with a Compact GC4.0 (Global Analyser Solutions, Breda, The Netherlands), with two channels. CO_2_ was measured on channel 1, which was equipped with an Rt-QSBond precolumn and column. Channel 1 used an injection split flow of 2 mL/min, temperature of 70°C, and pressure of 110 kPa. Channel 2 was equipped with a Molsieve 5A pre-column and Porabond Q column (to measure O_2_ and N_2_). O_2_ and N_2_ were characterized using an injection split flow of 10 mL/min, temperature of 70°C, and pressure of 100 kPa. Helium was used as carrier gas for both channels, and the column temperature was set to 50°C. All gases were quantified with a thermal conductivity detector set to 110°C with filament temperature of 210°C and a reference flow of 100 kPa.

#### Total CO_2_ production estimation

The CO_2_ concentration in the system or total inorganic carbon was estimated using the ideal gas law, Henry’s law, and the dissolution constants of CO_2_ (equations 5-12). The total inorganic carbon in the system (C_inorganic_ in mmol C/L) was estimated as the sum of CO_2_ in the headspace and all dissolved inorganic carbon species (i.e., [CO_2_]_aq_, H_2_CO_3_, HCO_3_^-^, CO_3_^2-^). The concentrations of dissolved species were derived from the headspace concentration assuming equilibrium between headspace and liquid according to the Henry constant, as well as equilibrium between all dissolved species according to the established dissociation constants (where *P* is the total absolute pressure in the system [atm], pCO_2_ is the partial pressure of CO_2_ [atm], m[CO_2_]_h_ is the measured concentration of CO_2_ in the headspace (%), [CO_2_]_h_ is the converted concentration of CO_2_ in the headspace [mol/L], [CO_2_]*_aq_* is the concentration of dissolved CO_2_ [mol/L], *T* is temperature [K], *V_h_* and *V_l_* is headspace and liquid volume [L], respectively, *n* is amount of substance [mol], *R* is the ideal gas constant, *k* is Henry’s constant, and *K_H_, K_a1_,* and *K_a2_* are the dissolution constants of CO_2_):


(5) $$pCO_{2}\;=\frac{m[CO_{2}{]}_{h}}{100}P$$

(6) $$pV=nRT;R=\;0.082\left(\frac{L}{K}\right)$$

(7) $$[CO_{2}{]}_{h}=\frac{pCO_{2}}{RT};T=301.15\;K$$

(8) $${\left[CO_{2}\right]}_{aq}=\;pCO_{2}\;k;\;k=\;3.4\times 10^{-2}\;\left(\frac{mol}{L\times atm}\right)$$

(9) $$\left[H_{2}CO_{3}\right]=\;{\left[CO_{2}\right]}_{aq}\;K_{H};\;K_{H}=1.7\times 10^{-3}$$

(10) $$\left[HCO_{3}^{-}\right]=\;\frac{\left[H_{2}CO_{3}\right]\;K_{a1}}{10^{-pH}};\;K_{a1}=2.5\times 10^{-4}\;\left(mol\;/L\right)$$

(11) $$\left[CO_{3}^{2-}\right]=\;\frac{\left[HCO_{3}^{-}\right]K_{a2}}{10^{-pH}};\;K_{a2}=4.7\times 10^{-11}\left(mol\;/L\right)$$

(12) $$\left[C_{inorganic}]\;=[CO_{2}{]}_{h}\frac{V_{h}}{V_{l}}+\;[CO_{2}\;{]}_{aq}+[H_{2}CO_{3}]+[HCO_{3}^{-}]+[CO_{3}^{2-}\right]$$

#### Specific rate of O_2_ consumption

The O_2_ consumption per cell (mmol/L/cell), or specific rate of O_2_ consumption, allows for a better comparison between bacterial species in different initial conditions and provides information on cellular activity (29). It was calculated using the following formula:


(13) $$O_{2}\;per\;cell\;=\frac{{\left[O_{2}\right]}_{t1}-{\left[O_{2}\right]}_{t2}}{\frac{Cell\;count{\;}_{t1}+Cell\;count{\;}_{t2}}{2}}\;$$

where the O_2_ concentration in the headspace ([O_2_] in mmol/L) between *t*_1_ and *t*_2_ is divided by the average total intact cell count between the two time points.

#### Thermogravimetric analysis

TGA was done in a TGA-1000-U (Navas Instruments, USA) for lime mortars. The whole sample (3.9 ± 0.2 g) was inserted into the machine and heated in a ceramic crucible in a N_2_ atmosphere, at 5°C min^−1^, from 20 to 1,000°C. The mass loss was converted to percentage by normalizing to the initial total mass. To calculate the Ca(OH)_2_ content in the sample (CH), the percentage weight (wt %) loss between 350 and 550°C (WL_CH_), molecular weights of Ca(OH)_2_ (74 g/mol), and water (18 g/mol) were used (equations 14 and 15). To determine the amount of CaCO_3_ in the sample (CC), the percentage weight loss between 650 and 950°C (WL_CC_), molecular weights of CaCO_3_ (100 g/mol) and CO_2_ (44 g/mol) were used (equations 16 and 17). Finally, from these values, the percentage weight of the converted Ca(OH)_2_ (CH_conv_) was estimated, then the total initial Ca(OH)_2_ (CH_i_) was calculated, and finally, the carbonation percentage (Carb) (%) was calculated (equations 18-20):


(14) $$WL_{CH}=\;wt{\%}_{350}-\;wt{\%}_{550}$$

(15) $$CH=\frac{WL_{CH}}{18}\times 74$$

(16) $$WL_{CC}=\;wt{\%}_{600}-\;wt{\%}_{950}$$

(17) $$CC=\frac{WL_{CC}}{44}\times 100$$

(18) $$CH_{conv\;}=\frac{CC}{100}\times 74$$

(19) $$CH_{i\;}=CH_{conv\;}+\;CH$$

(20) $$Carb=\frac{CH_{conv\;}}{CH_{i\;}}\times 100$$

### Carbonation of lime pastes

To determine the effect of bacterial suspensions in lime, lime pastes were prepared. Lime pastes were then tested at 7, 12, 15, 21, and 35 days using TGA, Fourier-transform infrared spectroscopy (FTIR), and phenolphthalein spraying.

#### Preparation of lime pastes

The bacterial isolates were grown in TSB, and a 1:100 inoculation was made in fresh TSB. The isolates were grown for 24 h to achieve full growth. The TSB suspension was centrifuged at 1,500 RCF for 10 min and resuspended in TSB without buffering agent (TSB-C: Pancreatic Digest of Casein 17.0 g/L, Peptic Digest of Soybean 3.0 g/L, Glucose 2.5 g/L, NaCl 5.0 g/L). Lime paste mixtures with either the resuspended bacteria or only TSB-C were prepared to study the capacity of the bacteria to carbonate the lime paste directly. Hydrated lime CL 90-S was used to prepare pastes using a 1:1 water-to-binder mass ratio, where the resuspended bacterial suspensions or only TSB-C were used in place of water. The paste was mixed by hand until homogeneous and cast into silicone molds with wells of 1.4 × 1.4 × 1.0 cm^3^ (w × l × h). Due to the fragility of the pastes, 20 samples per mix were prepared to ensure that 3 samples were tested at each date. The molds were tapped 30 times to remove internal bubbles and cured until the time of testing at 21°C and 65% relative humidity (RH). Leaving the samples in the mold meant that the carbonation front moved in a 1D, top-down direction.

#### Thermogravimetric analysis

Three lime paste samples were ground using a mortar and pestle. About 50 mg of powdered sample was then used to perform TGA in a Netzsch STA 449 Jupiter TGA-DTA Analyzer. The temperature ranged from 20 to 1,100°C at a heating rate of 10°C/min in an inert nitrogen atmosphere. The carbonation percentage was calculated as above.

#### FTIR spectroscopy

FTIR spectra of the precipitate were recorded on a NICOLET 20SXB FTIR (400–4,000 cm–1 spectral range; resolution of 2 cm^–1^).

#### Phenolphthalein analysis and measurement

A lime paste sample per mixture was split in half and sprayed with a 0.1% solution of phenolphthalein pH indicator in ethanol. The indicator solution was allowed to develop for 1 h, and images were taken of the samples through a microscope (Leica DMC 2900).

### Statistical analysis

Two-way analysis of variance (α = 0.05) was performed to examine the effect of bacterial isolates and time on the outcome variables (e.g., CO_2_ production, carbonation). Given significant differences in means (*P* < 0.05), we used Tukey’s honest significant difference test for *post hoc* pairwise comparisons to determine which isolate groups differed. All tests were performed in R.

## RESULTS

### Isolation campaign

An isolation campaign was carried out to find isolates with the goal of using them as carbonation enhancers in lime mortars. Given that lime has a high pH and hardens through incorporation of CO_2_, the isolates should be fit to grow at high pH and produce high amounts of CO_2_ at an early age. To ensure they were adapted to the environment, a lime mortar wall was chosen for the starting inoculum. A total of 33 isolates were obtained. MALDI-TOF MS (25) was used to dereplicate the isolates through the comparison of spectral differences, which left 16 reference strains remaining (Table S1). The most common genera were *Shouchella* (previously *Alkalihalobacillus*), *Bacillus,* and *Staphylococcus*. Finally, 16S rRNA gene sequencing was performed, and isolates were excluded based on their classification as biosafety level 2, leaving a final number of 12 isolates.

### Isolate selection

To find the isolates most adapted to high pH environment, growth experiments were performed in TSB pH 10 and then at pH 11. Alongside the 12 isolates, *Lynsinibacillus fusiformis* (LMG 9816) was used as a control, as it has been commonly used in other studies of MICP and is known to be an alkaliphile (30, 31). Only 5 of the 12 selected isolates grew successfully at TSB pH 10 (Fig. S4A), with *L. fusiformis* failing to do so, and were tested for their capacity to grow in TSB pH 11. Four isolates grew at pH 11 (Fig. S4B)—isolates 2 and 3 reached maximum OD within 48 h, while isolates 14 and 13 did so at 60 h and 75 h, respectively.

**Fig 2:**
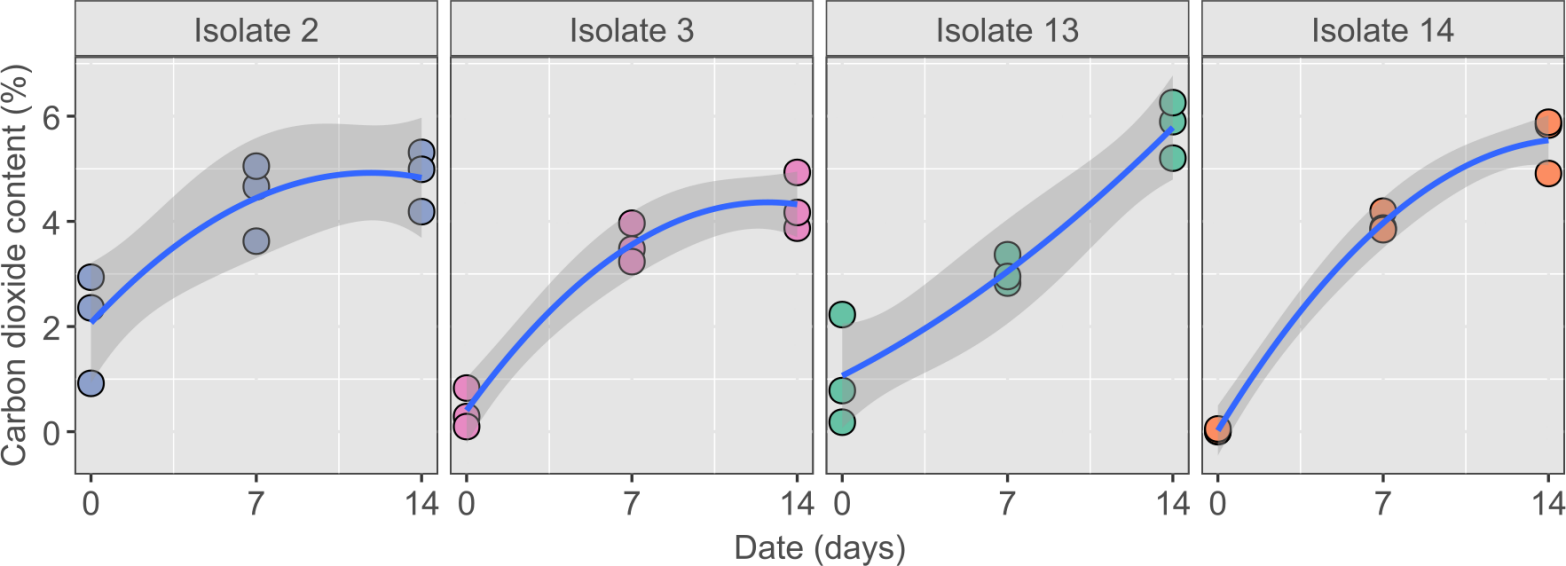
CO_2_ production (%) was measured at days 0, 7, and 14. Fully grown bacterial suspensions were used at day 0. TSB and water were used as controls (not shown). Isolates 2 and 14 produced the highest percentage of CO_2_ in the first 7 days, although isolate 13 caught up after 14 days. The best-fit curves indicate that CO_2_ production by isolates 2, 3, and 14 follows a logarithmic growth pattern, rising rapidly on the first days. The shaded area around the fitting curves shows a 95% confidence interval.

All four isolates were studied further for their CO_2_ production capacity to be used as carbonation enhancers of lime mortars. Here, a fully grown bacterial suspension in TSB was used as bacteria in the exponential phase will produce lower CO_2_ than those in the stationary phase (32). The fully grown suspension was centrifuged, and the pellet was resuspended in a fresh medium. The suspension was transferred to serum bottles, and CO_2_ concentration was monitored over time. Isolates 2, 3, and 14 could be grouped as fast CO_2_ producers, showing a logarithmic production of CO_2_ that rose rapidly at an early age (Fig. 2). Isolate 2 had a significantly higher CO_2_ production than isolate 3 (*P <* 0.05) but not than isolate 14. As such, isolate 3 was excluded from further study. The species-level classification after sequencing of isolate 2 was *Shouchella clausii* (LMG 33235), and isolate 14 was *Shouchella patagoniensis* (LMG 33400). These two isolates were the best adapted to high pH and were the fastest CO_2_ producers. They were therefore selected for a more in-depth characterization in a more realistic environment.

### Adaptive laboratory evolution for high pH tolerance

Given that lime has a pH of 12.4 in its freshest state, it can be a strenuous environment for bacteria to survive. Hence, an adaptive laboratory evolution experiment was performed to test whether it was possible to improve an isolate’s tolerance to the high pH, and if this could produce a more suitable isolate to use in the lime environment. *S. clausii* was chosen as it reached a maximum OD faster and higher than *S. patagoniensis* at pH 11 (Fig. S4), and it was hypothesized that a faster-growing isolate is more suitable to use in a high pH environment. *S. clausii* was grown repeatedly in TSB pH 11 for 10 transfers. Initial tests were done by mixing lime with TSB, but the medium would immediately precipitate, likely from denaturation of the media from the extreme pH range. Adjusting TSB with only NaOH was discarded due to the rapid drop in pH from a non-buffered system. Finally, TSB was adjusted to a pH of 11 using a CAPS buffer, which was the Good’s buffer with the highest pK_a_. The time for *S. clausii* to reach maximum OD was around 36 h (not shown), instead of the 48 h of Fig. S4 and S5, despite the same conditions. This was because the starting inoculum was a 1:100 dilution from a fully grown stock, rather than an inoculum with OD_600_ of 0.01 as used for the experiments shown in Fig. S4 and S5. The purpose here was to rapidly adapt the isolate to the high pH instead of comparing growth kinetics. After 10 transfers, the estimated number of generations was 34. A stock from the last transfer alongside the original stock was then grown in parallel in a second experiment. Gompertz curve fitting showed that the last generation isolate from the ALE experiment had a faster growth rate (*P <* 0.05) (Fig. S5). The isolate was deposited at the BCCM (LMG 33236) and is referred to as *S. clausii* transfer 10 (t10).

### Microbial activity in a high pH environment

After the selection of the isolates, a more comprehensive study was performed to study the response of the isolates to a high pH. This was performed in serum bottles with TSB adjusted to pH 11, and the consumption of O_2_ and production of CO_2_, alongside the media’s pH and the cell concentration, were followed. Fully grown bacterial suspensions were used for each isolate, and along the rest of the experiments, and these were centrifuged and resuspended in fresh medium prior to the experiment.

**Fig 3:**
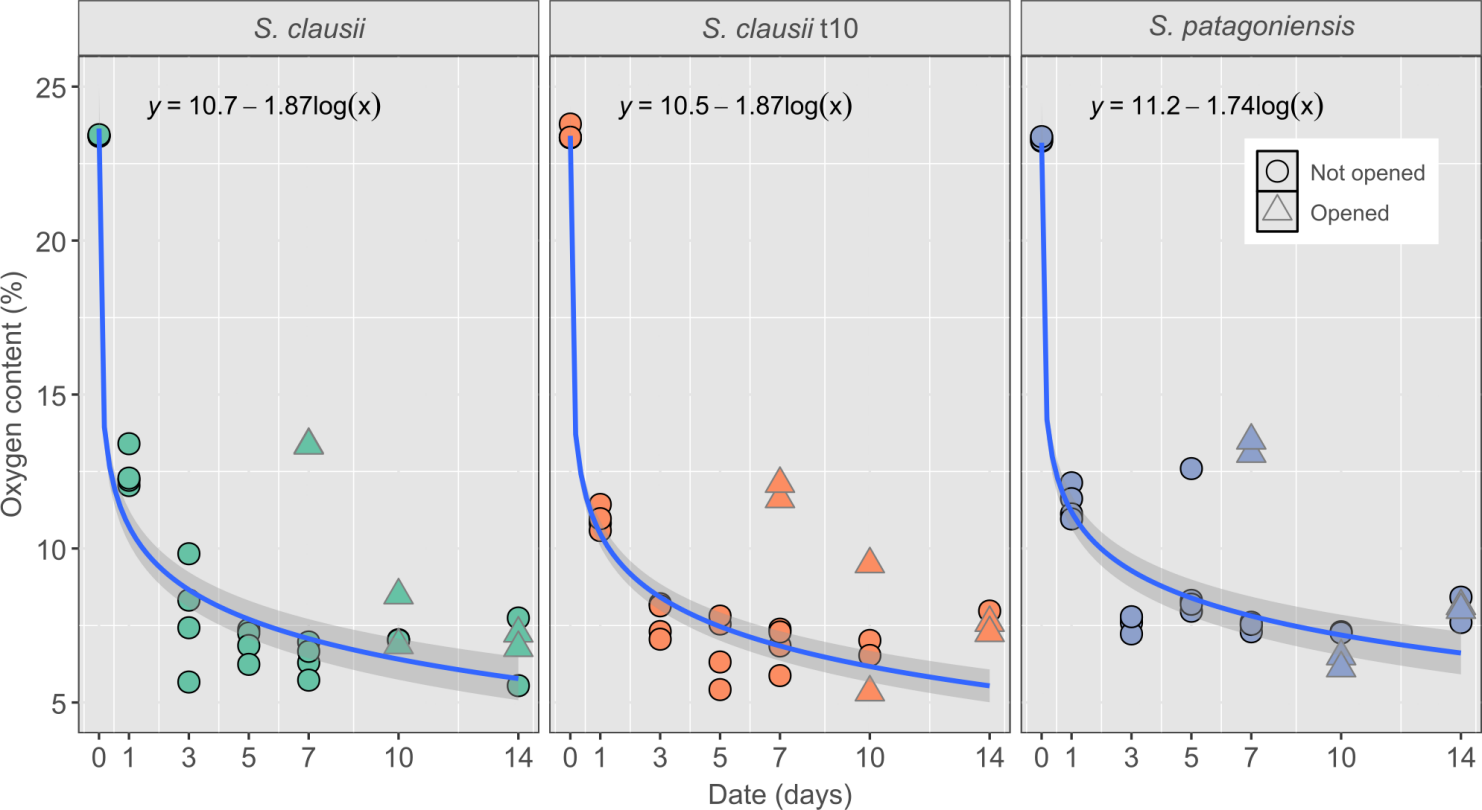
O_2_ content in the headspace of the serum bottles over time of the three tested bacteria*, S. clausii, S. clausii* t10, *and* S. *patagoniensis*. Fully grown bacterial suspensions were used at day 0. Two bottles were briefly opened at day 7 (triangles with gray outline) which allowed the O_2_ content to increase, but O_2_ was readily consumed by day 14. All controls showed a much more stable O_2_ content (Fig. S3). Regressions were performed on non-opened bottles. The shaded area around the fitting curves shows a 95% confidence interval.

All the isolates showed a logarithmic consumption of O_2_, and *S. patagoniensis* was the fastest consumer based on the regression model, albeit not significantly (*P* > 0.05) (Fig. 3). For all isolates, the O_2_ levels rapidly declined within 7 days to a minimum baseline of 7.22 ± 0.2%. To test the bacterial requirements for O_2_, at day 7 and once O_2_ consumption had seemingly stopped, two bottles from each condition were briefly opened, and the internal atmosphere was equilibrated with the external atmosphere (Fig. 3, gray outlined triangles). This resulted in an increase in O_2_ levels which were then rapidly reduced after 3 days. This shows that the system approached anoxic conditions. Nevertheless, when O_2_ became available, the bacteria were still capable of increasing their activity again.

As expected from the consumption of O_2_, the bacteria produced CO_2_ through oxidation of organic compounds, e.g., glucose and/or amino acids in the TSB. Nevertheless, the percentage of CO_2_ was unmeasurable for most of the experimental campaign. This was attributed to the pH of the media, which acts as a CO_2_ sink. Due to the high pH, CO_2_ would first dissolve in the media and accumulate as bicarbonate and carbonate species before headspace CO_2_ concentrations, above the limit of detection, would be reached. Therefore, CO_2_ was also indirectly measured following the pH of the media, which started at 11 and by day 14 dropped to between 9 and 10 in all cases, except for the control where the pH remained at a high stable value (Fig. S6). By day 14, there was a measurable concentration of CO_2_ in the headspace of *S. patagoniensis* samples and in one *S. clausii* t10 sample, but not in any *S. clausii* sample (Table 1).

**TABLE 1:** CO_2_ values and dissolved inorganic carbon in the system at day 14

Sample^*a*^	#	Opened at day 7	m[CO_2_]_h_^*b*^ (%)	pCO_2_ (atm)	[C_inorganic_]^*c*^ (mmol/L)
*S. patagoniensis*	4	Yes	1.0937	0.0091	160.80
*S. patagoniensis*	3	Yes	0.4783	0.0042	88.34
*S. patagoniensis*	2	No	0.2396	0.0013	93.44
*S. patagoniensis*	1	No	0.3884	0.0026	149.77
*S. clausii* t10	4	Yes	0.0325	0.0003	6.87
*S. clausii* t10	3	Yes	0.0000	0.0000	0.00
*S. clausii* t10	2	No	0.0000	0.0000	0.00
*S. clausii* t10	1	No	0.0000	0.0000	0.00

^*a*^ No CO_2_ was measured for *S. clausii*. Detailed calculations can be found in Table S3.

^*b*^ CO_2_ concentration was measured using gas chromatography.

^*c*^ Values calculated using the ideal gas law, Henry’s law, and the dissolution constants of CO_2_.

Due to the dissolution of the CO_2_ in the media, the headspace measurements did not provide the real total amount of inorganic carbon in the system. Therefore, the CO_2_ concentration was estimated using the ideal gas law (equations 4-11) in a similar fashion as done by reference 33. The estimated amount of total inorganic carbon in the system by day 14 ranged from 88 to 160 mmol C/L for *S. patagoniensis,* while opening or closing the bottles did not lead to a significant change (Table 1), despite that all opened bottles showed a lower pH than closed bottles for *S. patagoniensis*. The only *S. clausii* t10 replicate that had a measurable amount of CO_2_ was 10 to 100 times lower (6.87 mmol C/L).

The cell concentration throughout the experiment was measured with flow cytometry at 0, 3, 7, and 14 days (Table S2). Before the start of the experiment at day 0, all isolates were grown in the same initial conditions until a fully grown bacterial suspension was achieved, and the suspension was centrifuged and resuspended in fresh media. Despite these constant initial growth conditions, at day 0, *S. patagoniensis* had a significantly higher intact cell concentration (7.4 × 10^5^ cells/mL) than the other isolates (*P* < 0.05). *S. clausii* t10 had more than twice the cell number of *S. clausii* (5.4 × 10^5^ vs 2.0 × 10^5^ cells/mL, respectively), and the difference was significant. This was the main observable difference between the original *S. clausii* isolate and *S. clausii t10*. In the first 7 days, the intact cell population had little variation, but by day 14, there was a drop in cell concentration in all cases (2.3 × 10^5^, 1.8 × 10^5^, and 6.1 × 10^4^ cells/mL for *S. patagoniensis*, *S. clausii* t10*,* and *S. clausii,* respectively). Through the whole experiment, *S. patagoniensis* and *S. clausii* t10 had significantly higher amounts of intact cells than *S. clausii* (*P* < 0.05). On the other hand, the damaged population experienced a sharp drop by day 3 and then increased until day 14. In both these cases, *S. patagoniensis* had a higher damaged cell population than the other isolates (*P* < 0.05). The drop from day 0 to day 3 is likely due to all the initial damaged cell populations from the resuspended bacteria continuing to break down into debris, no longer being measurable at day 3. Then, as O_2_ got depleted (Fig. 3), and the fresh media got spent, the intact cells began to reduce and the damaged cell count increased (Table S2). We can see the exact opposite effect for the opened bottles, where there was a rise in cell count at day 14 for *S. clausii* t10 and *S. clausii* (open: 2.6 × 10^5^ and 1.5 × 10^5^ cells/mL vs closed: 1.8 × 10^5^ and 6.1 × 10^4^ cells/mL, respectively) likely from the increase in activity due to O_2_.

Opening the bottles led to a decrease in intact cells for *S. patagoniensis* (open: 1.7 × 10^5^ vs closed: 2.3 × 10^5^ cells/mL at day 14) (Table S2). Interestingly, there was a larger increase of damaged cells observed for *S. patagoniensis* in the opened bottles than in the closed ones (open: 3.9 × 10^5^ vs closed: 2.4 × 10^5^ cells/mL). This likely means that the increase in damaged cells was due to a rapid recovery of the cell population, where the intact population began to replicate, reaching a stationary phase and finally a death phase where cells began to degrade, all between day 7 and 14. This increase was not measured in the intact cell count at day 14 due to the time between measuring steps, but can be inferred from the flow cytometry density plots at day 14 by comparing intact and damaged populations (Fig. S7). The damaged cells in the plots of opened samples are denser and clustered together, suggesting a recent damage, and likely death, of the cells as the cells are still relatively intact. In contrast, the closed samples had a long cloud of red fluorescence, extending to the background, associated with different degrees of cell degradation and debris. This could further explain why the opened bottles of *S. clausii* did not have an increase in damaged cells, unlike its intact cells. Given the overall lower rate of O_2_ consumption and CO_2_ production from *S. clausii,* we can assume that the replication rate for *S. clausii* is slower, and, while *S. patagoniensis* cells have replicated and died, *S. clausii* are only just replicating. As such, *S. clausii* t10 replicates faster than *S. clausii,* but slower than *S. patagoniensis.*

**Fig 4:**
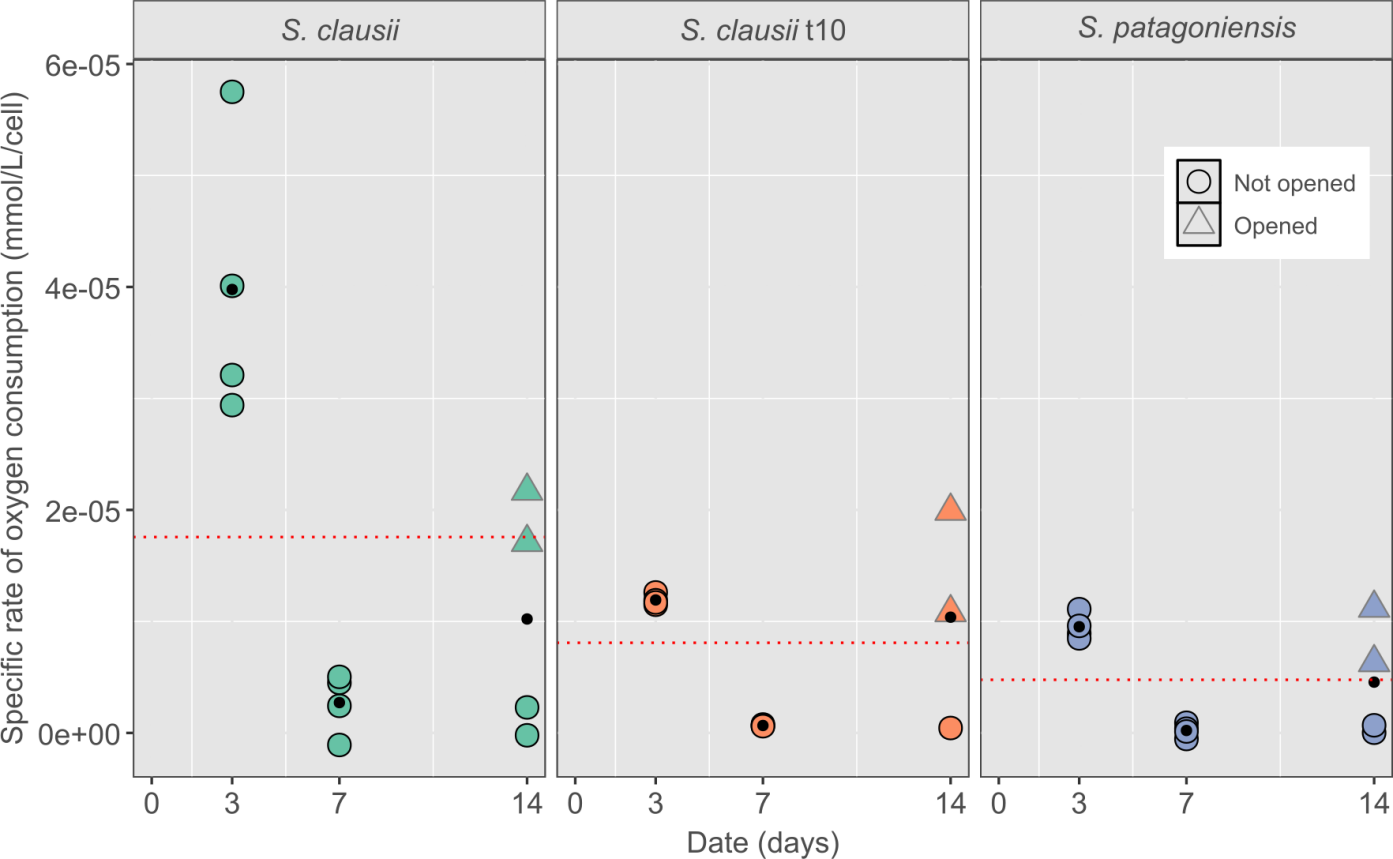
The specific rate of O_2_ consumption (mmol/L/cell) was calculated to observe the general O_2_ consumption of each species. O_2_ consumption can be directly related to the production of CO_2_. Triangles with gray outlines indicate bottles opened at day 7. Black dots indicate average for the date. Red dotted line indicates average for each species, and *S. clausii* had significantly higher consumption than the other two isolates *(P <* 0.05)*.*

The O_2_ consumption per cell or specific rate of O_2_ consumption (Fig. 4) was calculated to determine the metabolic activity of each species (29). On average, *S. clausii* is the biggest O_2_ consumer, most starkly observed in the first days of the experiment (*P* < 0.05). By day 7, *S. clausii* t10 and *S. patagoniensis* are no longer consuming O_2_, while *S. clausii* still shows activity. Overall, high specific rates of O_2_ consumption are associated with higher metabolic rates, and it is possible that the *S. clausii*, as it was the least adapted to the high pH environment, was spending more energy. This could also explain the limited total cell numbers, as it was not in an optimal environment. For all isolates, opening the samples at day 7 led to an increase in O_2_ consumption per cell by day 14.

### Carbonation of lime mortar samples** **

**Fig 5:**
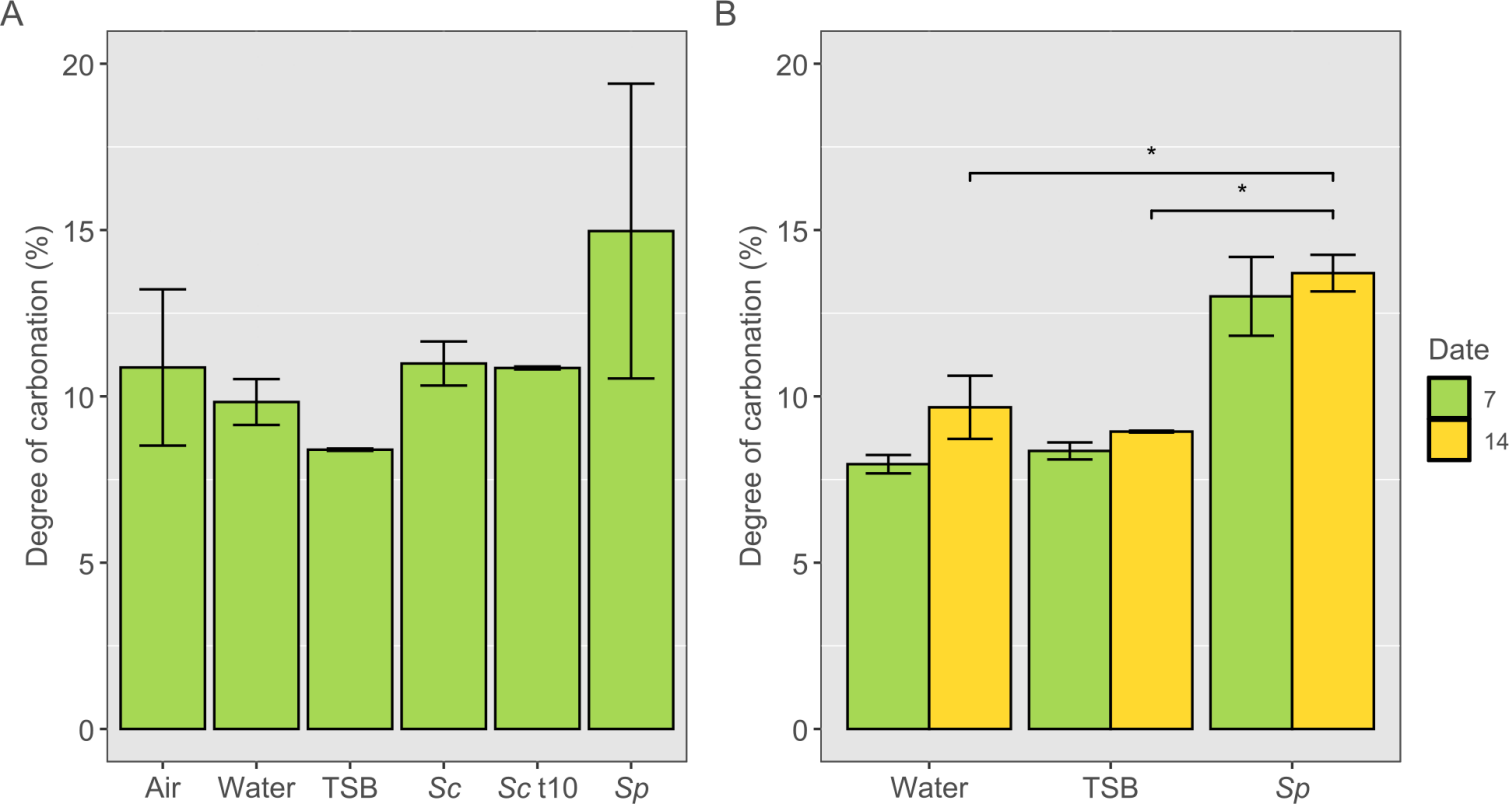
Degree of carbonation of lime mortar samples for different suspensions tested up to 7 days (*n* = 4) (**A**) and replicate tested up to 14 days (*n* = 6) (**B**). The cottons were soaked with water (Water), TSB (TSB), *S. clausii* (*Sc*), *S. clausii* t10 (*Sc* t10), and *S. patagoniensis* (*Sp*)*.* * indicates a significant difference (*P <* 0.05)*.* Error bars indicate standard error. Thermogravimetric curves can be found in Fig. S8. No significant difference was observed between the conditions (*P >* 0.05)*.*

Although isolates were selected for their CO_2_ producing capacity, it was necessary to test whether carbonation of lime was possible with the bacterial suspensions. To do so, lime mortar samples were carbonated in an enclosed environment with cotton soaked in fully grown bacterial suspensions in fresh media from each isolate. The samples were incubated for 7 days, at which point the experiment was terminated. All samples had a baseline of carbonation as seen on all three controls (Fig. 5A), likely arising from the preparation process of the mortar samples. Nevertheless, the *S. patagoniensis* suspension seemed to have further carbonated the samples to a higher degree than the controls of air, water, and TSB, although this difference was not significant (*P* > 0.05).

To increase confidence in the effect of bacterial carbonation results, the experiment was repeated using *S. patagoniensis* and extended to 14 days. Once again, a baseline carbonation is observed on the Water and TSB controls, and *S. patagoniensis* also led to a significantly higher carbonation than both (*P* < 0.05) (Fig. 5B). The extent of carbonation was consistent with the first experiment (Fig. 5A). By 14 days, no further progression of carbonation seems to have taken place in the system, indicating a saturation of the carbonation capacity.

### Bacterial lime pastes

**Fig 6:**
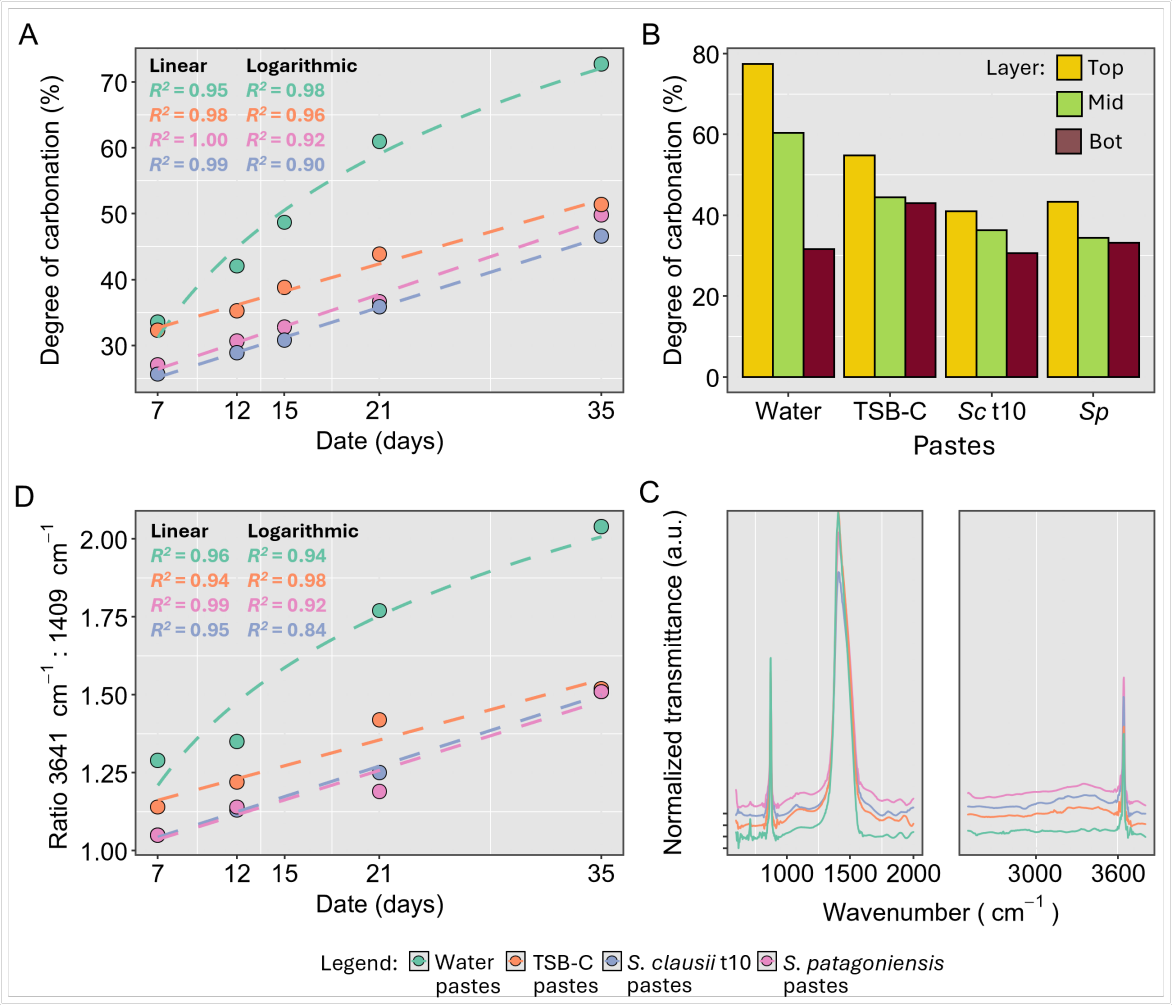
Lime paste samples with water (green), TSB-C (orange), *S. clausii* t10 (blue), and *S. patagoniensis* (purple) were tested at 7, 15, 14, 21, and 35 days using TGA, FTIR, and phenolphthalein analysis. In clockwise order: (**A**) degree of carbonation of lime pastes determined by TGA. *R*^2^ values for linear and logarithmic regressions of the progression of carbonation of all pastes. TSB-C and bacterial pastes have a better fit with linear models, while water’s best fit is logarithmic. TGA curves can be found in Fig. S9A. (**B**) Degree of carbonation for the separate layers of the sample top (yellow), middle (mid, green), and bottom (bot, red). TGA curves can be found in Fig. S9B. (**C**) FTIR spectra of the lime pastes at day 15 between 600 and 2,000 cm^−1^ and 2,700 and 3,700 cm^−1^, where organic signatures were detected. Given that one sample per paste and date was tested on all measurements, no statistical analyses were performed. (**D**) Ratio between the absorbance of OH band (3,641 cm^−1^) of Ca(OH)_2_ and the v_3_ mode of CaCO_3_ at 1,409 cm^−1^*.*

*S. patagoniensis* and *S. clausii* t10 were chosen to perform experiments in which they were directly mixed into lime pastes. Fully grown bacterial suspensions were used for both, and these were centrifuged and resuspended in fresh TSB-C. While *S. patagoniensis* was the best-performing isolate from the results of the carbonation of the lime mortar samples, *S. clausii* t10 was seen to grow better at high pH and was therefore also examined. The evolution of the carbonation in the pastes was followed by TGA, FTIR, and phenolphthalein spraying, and the lime pastes were tested at 7, 12, 15, 21, and 35 days (Fig. 6). Pastes were also prepared by mixing lime with water and TSB-C to act as controls and determine the effect of bacteria addition.

TGA indicated that the water-based pastes achieved the highest carbonation percentage (Fig. 6A). It followed a logarithmic relationship to time, as expected in these systems (10). The other pastes—TSB-C paste, *S. clausii* t10 paste, and *S. patagoniensis* paste—instead did not, but carbonated linearly with respect to time, indicating a different carbonation process (Fig. 6A). Overall, TSB-C pastes carbonated faster than the bacterial pastes at the beginning, matching the rate of the water paste controls at day 7, but by 35 days, the bacterial pastes had an increased rate of carbonation, matching the carbonation degree of TSB-C pastes. The TGA curves for the water paste controls show the expected behavior, with predominant mass losses between 350 and 550°C (corresponding to portlandite) and from 600 to 950°C (corresponding to calcium carbonate). TSB-C and bacterial pastes showed a third mass loss between 25 and 200°C, likely from organic matter (Fig. S9A).

At day 21, the pastes were separated into three layers (top, middle, and bottom) and ground to evaluate the progression of carbonation throughout the sample’s profile (Fig. 6B). In all cases, the top layer was the most carbonated. In the pastes with water, the mid-layer was also more carbonated than the bottom layer. For TSB-C and bacterial pastes, these two lower layers were similarly carbonated. Remarkably, for the TSB-C paste, the bottom layer had more carbonation than that of the water paste, showing that carbonation progressed more evenly on these samples than the control. For the bacterial pastes, the bottom layer was equally carbonated as the pastes made with water only, meaning no effect was measurable. All TSB-C and bacterial layers also showed a weight loss during TGA between 25 and 250°C, coming from the presence of organic material (Fig. S9B).

FTIR confirms the presence of organic phases, present as a shoulder between 1,500 and 1,700 cm^−1^ and as a broad band between 3,000 and 3,600 cm^−1^ (Fig. 6C). These were ubiquitous in TSB-C and bacteria-containing pastes. Moreover, in all cases, characteristic *v*-modes of calcite were observed (712, 874, and 1,409 cm^−1^) and a strong OH stretching (3,641 cm^−1^), corresponding to portlandite. Interestingly, the development over time of the ratio between the portlandite peak vs the main calcite band (1,409 cm^−1^) was remarkably similar to that of the percentage of carbonation determined using TGA (Fig. 6D). The v_3_ mode has been used to determine carbonation extent in cement-based materials (34).

A standard progression of carbonation was observed following phenolphthalein application for pastes with water, where a whiter (more carbonated) region progressed downward to the bottom, less carbonated (pink) region of the sample, along the direction of carbonation (Fig. S10). Instead, for TSB-C and bacterial pastes, a two-step carbonation progression was observable. Here, a slow drop in pH of the lime pastes occurred, whereby at 7 days, the carbonation front was not visible. At 12 days, whiter regions were observable in the TSB-C and bacterial pastes, but only at day 15 was there a visible white band. This carbonated region was centered in the sample, in between two pink bands of uncarbonated lime, a thin one on the surface and a thicker one at the bottom, showing that the carbonation front progressed in a non-uniform, discontinuous way (Fig. S10). Conversely, when the samples were tested at day 21, we observed that the carbonation front was measurable again and that the surface band of dark pink disappeared. At day 35, this phenomenon repeated, a lighter center indicating carbonation was surrounded by pink bands, although slight variations per samples were visible. This phenomenon was observable when the experiments were repeated.

## DISCUSSION

The focus of this work was to present a pilot experiment on the use of bacteria to improve the carbonation of lime-based materials by producing CO_2_ to speed up the process (equation 1). This would help lime mortars set faster at an early age. Due to the high pH of lime (12.4 in fresh state), only alkaliphilic bacteria were deemed suitable to use here. An isolation campaign was conducted from an old lime mortar in a masonry wall, and isolates were selected based on their alkalophilicity and CO_2_ production capacity, while further characterization of their O_2_ consumption rates and cell numbers was performed. Finally, the capacity for bacterial CO_2_ to carbonate lime mortars was tested, and the impact of mixing bacterial suspensions with lime on the carbonation process was examined.

Thirty-three isolates were isolated from a lime mortar wall and were characterized through MALDI-TOF and sequencing to obtain 16 distinct isolates, 12 of which were non-pathogenic. All genus results were expected, given the type of sample, and were mostly alkaliphilic. The most common ones*—Shouchella*, *Bacillus,* and *Staphylococcus*—are all part of the Firmicutes phyla, which is dominant in limestone environments (35, 36). *Shouchella* is one of the main *Bacillus* clades and is particularly known for being alkaliphiles (37). Four isolates showed a capacity to grow fast and effectively at pH 11, which is one of the highest pHs achieved with Good’s buffer solutions. From these four isolates, three were fast CO_2_ producers, and the best two were selected for further study, namely *S. clausii* and *S. patagoniensis* isolates. *S. clausii* was further grown in a high pH environment in an adaptive laboratory evolution experiment. *S. clausii* was chosen over *S. patagoniensis* due to its better growth kinetics, under the hypothesis that these mean better adaptation to high pH, and adaptation is linked to CO_2_ production (38). The isolate was grown over 10 transfers in TSB pH 11 and a total of around 34 generations. The isolate from the final transfer, *S. clausii* t10*,* was observed to have a faster growth rate than *S. clausii* and higher stationary phase cell density at pH 11, observed through cell counts*.* We consider the higher cell counts for *S. clausii* t10 indicative of low stress. This is because more energy can be invested into replication rather than the high energy needs for cells to maintain homeostasis or other non-growth-associated metabolic activities due to suboptimal environment (32). In turn, this leads to an increase in biomass at stationary phase (32, 38, 39), coinciding with what is observed in the results presented here. The faster growth rate and higher cell count were an indicator that the adaptive laboratory evolution of *S. clausii* was partly successful and led to acclimation of *S. clausii* t10 to high pH. The number of generations, 34, is on the lower end of what is common in adaptive laboratory evolution (40), and extending the length of the experiment would have possibly provided an even larger difference in growth rate.

The growth dynamics and capacity for carbonation of lime mortars of all three isolates, *S. clausii, S. clausii* t10, and *S. patagoniensis,* were then studied in parallel experiments. A fully grown suspension was used as starting inoculum, as replicating bacteria will produce less CO_2_ than those in stationary phase (32). We hypothesized that stationary phase cells are not replicating as frequently, and so biosynthetic pathways are less active, and carbon is not incorporated into macromolecules. Given that the objective is to achieve better carbonation, where higher CO_2_ concentrations are desired, then stationary phase activity was preferable. The use of a fully grown suspension also ensured that there was no contamination or competition by possible indigenous species, due to the high cell density outcompeting any possible colonization of the nutrients. The experimental series following microbial activity in serum bottles showed that *S. patagoniensis* had the highest intact cell count of all isolates when grown in TSB pH 11 and had a higher replication rate between days 7 and 14 than the other isolates. Moreover, *S. patagoniensis* was the fastest consumer of the O_2_ in the headspace of the bottles. Even when the samples were opened on day 7 to introduce more O_2_, *S. patagoniensis* was the quickest to increase its activity and deplete the available O_2_, increasing cell counts faster than the other two isolates. Additionally, *S. patagoniensis* exhibited the lowest specific rate of O_2_ consumption. Since the specific rate reflects metabolic activity, a lower demand for O_2_ indicates a lower organic acid consumption, i.e., lower energy demand (41, 42). This lower energy demand could be attributed to a better acclimation or even adaptation to the high pH environment. Overall, *S. patagoniensis* seemed the best adapted to the high pH, outperforming *S. clausii* and *S. clausii* t10. Lastly, *S. patagoniensis* was the only isolate that produced a measurable amount of CO_2_ in the headspace of the serum bottles, which confirms that it was the best CO_2_ producer of the studied isolates. Unfortunately, CO_2_ in the headspace could only be measured at day 14, and the evolution of CO_2_ production could not be followed. This was due to the high pH environment in which tests were conducted, which acted as a carbon sink, and it was only after pH reached 9–9.5 that CO_2_ began to accumulate in the headspace. Nevertheless, it is possible to correlate the aforementioned cell density and growth phase, as well as inversely correlate the consumption of O_2_, of *S. patagoniensis* with the production of CO_2_ (32).

The specific rate of O_2_ consumption also allows comparing species in different starting conditions (43). Here, the cell count differed per species, but the starting concentrations in each experiment were not altered as species-specific differences in similar starting conditions were of interest in determining the most suitable isolate. Through the use of O_2_ consumption per cell, it is possible to see that the higher cell count of *S. patagoniensis* accounted for the larger CO_2_ production of all three isolates. This contradicts our initial hypothesis that a more adapted strain will more readily produce CO_2_, but it is possible that the high pH stress on *S. clausii* is leading to an even higher increase in metabolic activity as more energy is needed to maintain homeostasis. Such cases have been observed on other organisms in the presence of excess carbon but limiting nutrient deficiencies (44). Furthermore, a higher specific rate of O_2_ consumption also implies that the least adapted strain will have a lower CO_2_ production but a higher yield (i.e., the production per unit). As such, depending on the optimal concentration of cells needed to carbonate lime, a different strategy to that hypothesized here might become more suitable: if a lower cell count is possible, a less adapted strain with a higher CO_2_ yield would be a more optimal carbonation solution.

The measurements of CO_2_ at day 14 also allowed us to estimate the total amount of CO_2_ produced by the bacteria that was present as CO_2_ in the headspace, CO_2_ dissolved in the media, and CO_2_ converted to H_2_CO_3_, HCO_3_^-^, and CO_3_^2-^. The amount of CO_2_ produced by *S. patagoniensis* by day 14 was 88 to 160 mmol/L. This accounts for a CO_2_ production rate of 6 to 12 mmol/L/day or 6.94 × 10^−5^ to 1.39 × 10^−4^ mmol/L/s (assuming a constant production rate through the whole experiment, which is not the case). Following Van Balen and Van Gemert, where it is assumed that all CO_2_ will react with portlandite in a mix system (with excess Ca(OH)_2_), the production rate of CO_2_ by the bacteria becomes comparable to the values reported by these authors for CO_2_ uptake during carbonation (6). In this case, the upper limit of *S. patagoniensis* production capacity is relatively close to the lowest margin of the maximum CO_2_ uptake by lime in an ideal scenario (3.99 × 10^−4^ 1/s). Given that no optimization for maximizing CO_2_ production was performed on *S. patagoniensis*, and that these values are conservative, we can conclude that the use of bacterial CO_2_ might provide a viable and useful method for early age carbonation of lime. Extrapolating these results to a full-scale system would likely affect these rates, though. Lastly, when looking at the carbonation of the lime mortar samples in a closed atmosphere, *S. patagoniensis* carbonated the samples faster and to a higher degree than the other two isolates. Still, in the case of lime mortar samples, a high variability was observed between runs, and the carbonation did not progress further after 14 days compared to 7 days. This was not the case in the serum bottle experiment, where CO_2_ was highest at 14 days. This is expected to be due to the different setups with which carbonation and CO_2_ measurements were tested.

To show the possibility of bacteria to be used as an earlier hardening agent of lime, we performed direct mixing of the bacteria suspensions with lime in pastes. The use of pastes allows for confirming whether the changes in the carbonation progress are more directly attributed to the bacterial additives and not to other interactions that can be caused by aggregates (45). Interestingly, an inhibitory effect was observed for the TSB-C and bacterial pastes when compared to the water controls, although it was accompanied by a very different carbonation dynamic. A two-step carbonation process was observed, whereby a lower pH (linked to the carbonation front) could be measured only after 12 to 15 days, depending on the mixture. Still, this region of carbonation was sandwiched between two uncarbonated bands closer to the surface and bottom of the sample, respectively. To explain the advance of the carbonation front past an uncarbonated region, we hypothesize that it was due to the development of a Liesegang pattern (45, 46). This type of pattern appears in porous systems subjected to diffusion-reaction processes, as is the case of a lime paste undergoing carbonation. The diffusion (porous) medium is the lime paste itself, and the diffusing reactant is CO_2_ toward the uncarbonated portlandite particles. Following carbonation, an amorphous calcium carbonate metastable nanophase forms, which can also diffuse in a partially saturated medium and eventually transform into a stable crystalline CaCO_3_ (calcite) at a particular location where a Liesegang band appears (i.e., lighter band surrounded by pink bands after phenolphthalein application). After this event, the carbonation front progresses to the sample core, where another precipitation event can take place forming another CaCO_3_ band (46). However, the dissolution of uncarbonated portlandite and redistribution of this solution along the pore space of the lime plaster can lead to a pH increase even in areas where carbonation has already taken place, resulting in the periodic bands associated with the Liesegang phenomenon, usually formed in rapidly carbonating samples (3). This would explain the more homogeneous carbonation observed in the different layers of the pastes mixed with TSB-C tested at 21 days, whereas the pastes mixed with water had a stratified carbonation. Interestingly, these Liesegang patterns have been associated with superior lime mortars (46). The change in carbonation progression in the TSB-C and bacterial pastes can certainly be attributed to organic phases found in these mixtures. Organic presence was confirmed by weight loss in the 25°C–250°C range using TGA and in the FTIR analysis, at the bands between 1,500 and 1,700 cm^−1^ and 3,000 and 3,600 cm^−1^.

Despite the promising effect of the Liesegang pattern formation seemingly induced by the organics, the overall carbonation was inhibited in these pastes. The use of TSB as a bacterial medium could have partly influenced this as it contains a concentration of 0.5% of NaCl. At higher concentrations (3.5%–7%), NaCl rapidly increases carbonation at an early age. This creates a dense carbonation layer at the surface that inhibits the carbonation process by 28 days in aerial mortars (47). On the other hand, TSB also contains glucose, which also has an inhibitory effect on carbonation, but this leads to the opposite effect in the long term compared with NaCl, where CO_2_ can diffuse through the whole sample, carbonating better inside and improving the mechanical properties of the materials (47). As such, TSB contains two compounds, although at low concentrations, that inhibit carbonation in the short term (28 days), which would explain the lower performance of the TSB-C and bacterial pastes compared to water. Finally, the bacterial biomass seems to further inhibit carbonation early on, but a ramp-up is experienced by day 35, achieving similar degrees of carbonation as the TSB-C control. It is possible that bacterial CO_2_ allowed for this improvement, as was seen in the lime mortar samples. Nevertheless, the length of the experiment did not allow us to observe any discernible bacterial effect, and the mechanistic explanation for this rate improvement at the end of the experiment is not possible to outline here.

This work was not an exhaustive analysis of the bacterial isolates, nor were the results optimal. Yet, it serves as a starting point for the use of bacterial CO_2_ as a strategy to improve carbonation in lime mixtures. Only two previous works have explored this possibility before, showing the topic is still in its infancy (15, 16). Importantly, our work expands on this previous research by looking in depth at a more robust selection process for isolates and correlates the production of CO_2_ with the carbonation of lime. Still, further work is required to prepare lime mortar formulations optimized for bacterial carbonation strategies. For example, how bacteria and organic additives lead to a linear carbonation progression needs to be more rigorously studied. Also, dissolved inorganic carbon was not measured, and such data would prove valuable to confirm that the drop in pH is only associated with respiration of carbon sources and not the influence of other organic acids. Moreover, the media composition should be carefully reassessed. In this work, TSB was used, which affected carbonation when mixed directly with lime, even though the buffering component was removed (TSB-C). Moreover, TSB-C had NaCl, which in future applications could lead to salt damage (48). TSB can also be too expensive at economies of scale, and the choice of media is central in determining the scalability of a product (49), while certain media components, particularly carbon sources like glucose, can have varying carbon footprints which need to be carefully assessed (50). Finally, further research should also focus on analyzing the mechanical and physico-chemical effect that the bacterial suspensions have on the ultimate performance of the lime mortars. Tests are required to determine whether, even with changes in carbonation, other durability and microstructural properties are influenced, and to compare this novel strategy to the most promising approaches to speed up carbonation of lime-based binders, such as the use of carbonic anhydrase and metal-organic frameworks (51). Note that the possible formation of carbonate-organic hybrids with superior physical-mechanical properties as in the case of carbonate biominerals in general (e.g., sea shells [52]) and bacterial biominerals in particular (24) would lead to better performance not only in terms of carbonation speed at early ages, but also on improved long-term durability of these bio-based lime mortars and plaster.

### Conclusion

This study was a proof of concept of the potential of bacterial CO_2_ production to accelerate the carbonation of lime mortars, offering a promising bio-based strategy for enhancing early-age strength. It expands on previous studies by providing a more systematic selection of bacterial strains and directly linking CO_2_ production to carbonation efficiency. Among the isolates, *S. patagoniensis* exhibited the best adaptation to high pH conditions and the highest CO_2_ production, directly contributing to improved carbonation on lime mortars when compared to the other studied isolate. Despite some promising results, practical aspects of bacterial delivery must be addressed as the results presented here were not successful when bacteria were mixed with lime directly (i.e., lime pastes). The variation in carbonation progression, specifically the development of Liesegang patterns, induced by the organics is promising, but additional mechanistic studies are needed to fully understand the interplay between bacterial additives and lime materials.

## Data Availability

The 16S rRNA gene sequences were deposited in the National Center for Biotechnology Information (NCBI) archive under GenBank accession numbers PV163962, PV163981, and PV163963. All other data needed to evaluate the conclusions in the paper are present in the paper and/or the supplemental material.

## SUPPLEMENTAL MATERIAL

Supplemental material aem.00683-25-s0001.pdf Figures S1 to S10; Tables S1 to S3.

ASM does not own the copyrights to Supplemental Material that may be linked to, or accessed through, an article. The authors have granted ASM a non-exclusive, world-wide license to publish the Supplemental Material files. Please contact the corresponding author directly for reuse.
