# Supplementary material for: Characterization of isolates used in bacterial-based strategies for accelerated carbonation of lime mortars
Source: Appl Environ Microbiol. 2025 Sep 23;91(10):e00683-25. doi: 10.1128/aem.00683-25 (PMC12542689; doi:10.1128/aem.00683-25)
Supplement: Supplemental material — Figures S1 to S10; Tables S1 to S3. [file aem.00683-25-s0001.pdf]

Appendix for the manuscript "Characterization of Isolates used in Bacterial-Based Strategies for Accelerated Carbonation of Lime Mortars"

Authors

Franco Grosso Giordano[a,b,c], Quinten Mariën[a], Nele De Belie[b], Carlos Rodriguez-Navarro[c] and Nico Boon[a]#

Email addresses

franco.grossogiordano@ugent.be; quinten.marien@ugent.be; nele.debelie@ugent.be; carlosrn@ugr.es; nico.boon@ugent.be

Affiliations

[a] Ghent University, Center for Microbial Ecology and Technology (CMET), Ghent, Belgium.

[b] Ghent University, Magnel-Vandepitte Laboratory, Dept. of Structural Engineering and Building Materials, Ghent, Belgium.

[c] University of Granada, Department of Mineralogy and Petrology, Granada, Spain.

# Corresponding author: nico.boon@ugent.be

Appendix

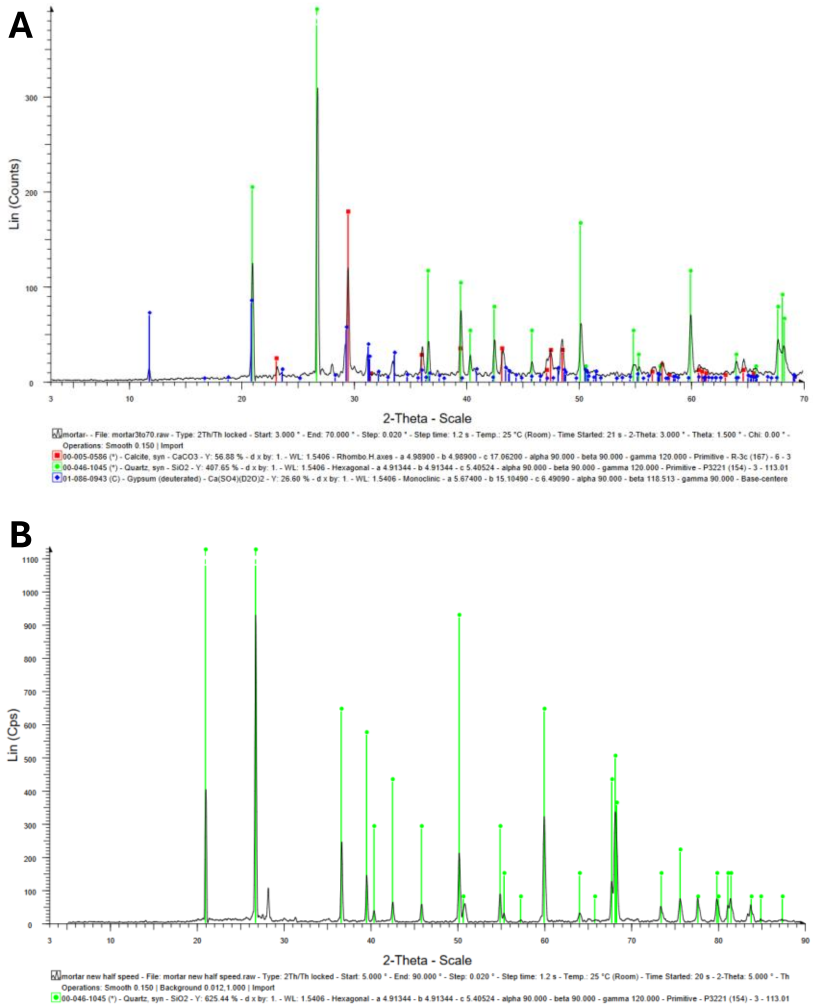

Figure S 1 – XRD patterns of a fragment of lime-mortar wall before (A) and after (B) acid attack by 37 % HCl solution. All Bragg peaks in A correspond to calcite and quartz with some indications of gypsum, while only quartz and small amounts of feldspar (Bragg peak at ~28 °2θ) are observed after the acid attack (B).

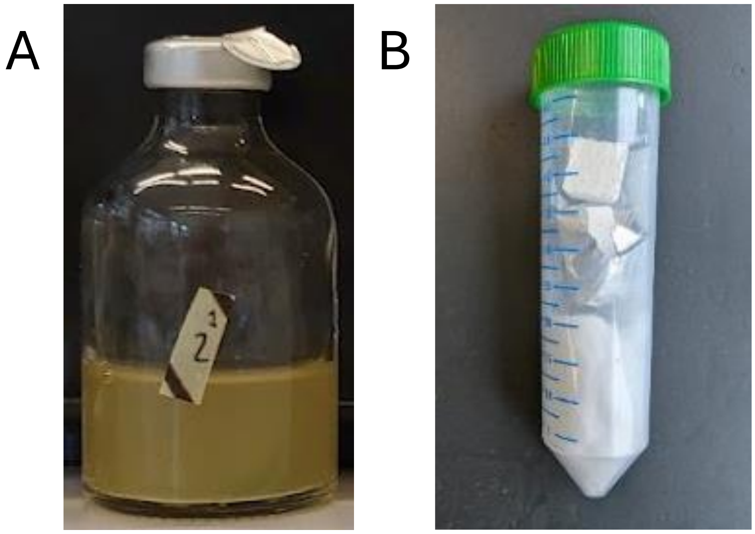

Figure S 2 – A) 30 mL serum bottle capped with butyl rubber stoppers and sealed, used for following metabolic kinetics of the different isolates including measuring gas production, cell counts and pH over time. B) Arrangement of the soaked cotton, aluminum paper and lime mortar samples inside a Falcon tube. This system acted as a closed environment to test carbonation under an atmosphere with increased CO_2 due to bacterial respiration.

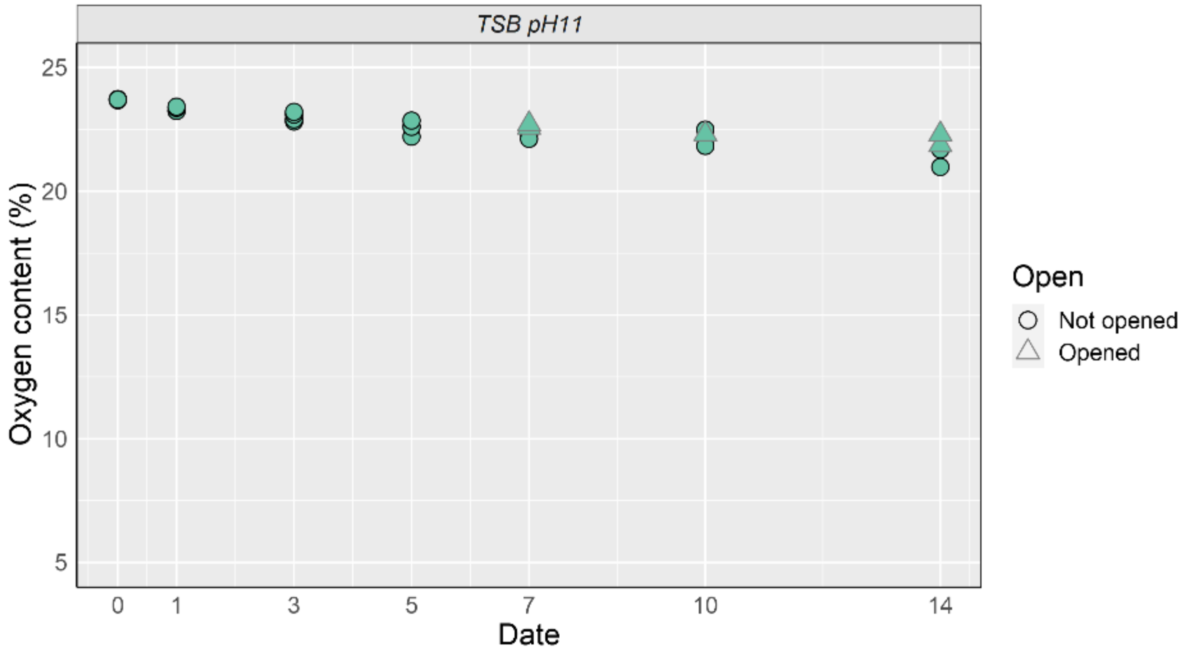

Figure S 3 – O_2 content in the controls*, bottle with sterile TSB*. The *O_2* level slowly decreases overtime *likely from sampling*. The bottles that were opened *(triangles with grey outline)* also did not see a change in *O_2* concentration, given that the

internal atmosphere remained at the same pressure so there was no active exchange of gas. *The addition of NaN_3 to one of the bottles shows no change in O_2 concentration, nor CO_2 (not shown).*

Table S 1 – All isolates from the isolation campaign according to the media on which they were isolated[1]

Isolation media	Isolate name	Reference group	Reference strain	MALDI-TOF MS hit	16S rRNA sequencing hit
R2A	R2A-3B*	A	0	*Bacillus sp.*	*Bacillus sp., cereus, thuringiensis, paranthracis, pacificus, toyonensis*
R2A	R2A-4F	A		*NA*	
**R2A**	**R2A-5F**	**B**	**1**	***Bacillus thuringiensis***	***NA***
**Horikoshi**	**Hori-4D**	**C**	**2**	***Bacillus clausii***	***Alkalohalobacillus clausii***
**Horikoshi**	**Hori-3B**	**D**	**3**	***Bacillus clausii***	***Bacillus velezensis, amyloliquefaciens, subtilis***
**R2A**	**R2A-4J**	**E**	**4**	***NA***	***Bacillus sp., mobilis, toyonensis, wiedmannii***
R2A pH 9	R2A9-H.B*	F	5	*NA*	*Bacillus mobilis, toyonensis or wiedmannii, cereus*
R2A	R2A-3A.A	G		*Bacillus sp.*	

R2A	R2A-3A.B	G		*Bacillus sp.*	
R2A	R2A-3C*	G	7	*Bacillus sp.*	*Bacillus thuringensis, cereus mobilis or wiedmanni*
R2A pH 9	R2A9-4H.A	G		*NA*	
**R2A pH 9**	**R2A9-4K**	**G**	**6**	***Bacillus sp.***	***NA***
**R2A pH 9**	**R2A9-3A**	**H**	**8**	***Bacillus thuringiensis***	***Staphylococcus warneri***
R2A	R2A-4H	I		*Staphylococcus warneri*	
**R2A pH 9**	**R2A9-3C.C**	**I**	**10**	***Staphylococcus warneri***	***Sphingomonas sp.***
**R2A pH 9**	**R2A9-4E**	**I**	**9**	***Staphylococcus warneri***	***Staphylococcus warneri***
R2A pH 9	R2A9-4F	I		*Staphylococcus warneri*	
R2A	R2A-4E	J		*NA*	
R2A pH 9	R2A9-3C.A*	J	11	*NA*	*Bacillus mobilis, toyonensis, wiedmannii, cereus*
**R2A pH 9**	**R2A9-3C.B**	**K**	**12**	***NA***	***Alkalobacillus sp, patagoniensis***

**Horikoshi**	**Hori-3C**	**L**	**14**	***NA***	***Alkalobacillus sp, patagoniensis***
Horikoshi	Hori-4E	L		*NA*	
**Horikoshi**	**Hori-5F**	**L**	**13**	***NA***	***Alkalobacillus clausii***
Horikoshi	Hori-5G	L		*NA*	
**R2A pH 9**	**R2A9-3B**	**M**	**15**	***Bacillus sp***	***Alkalobacillus sp, patagoniensis***
R2A pH 9	R2A9-5J	M		*NA*	
Horikoshi	Hori-3A	N		*NA*	
R2A	R2A-3D	NA			
R2A	R2A-4G	NA			
R2A	R2A-4I	NA			
R2A pH 9	R2A9-3D	NA			
R2A pH 9	R2A9-4G	NA			
R2A pH 9	R2A9-5I	NA			

[1]*Shown are the isolate name, the reference group according to MALDI-TOF MS analysis, the reference strain of each group according to MALDI-TOF MS analysis, the classification according to MALDI-TOF MS analysis and classification according to 16S amplicon sequencing. Bold rows are the 12 candidates selected after isolation for further analysis. * indicates strains that are biosafety level 2.*

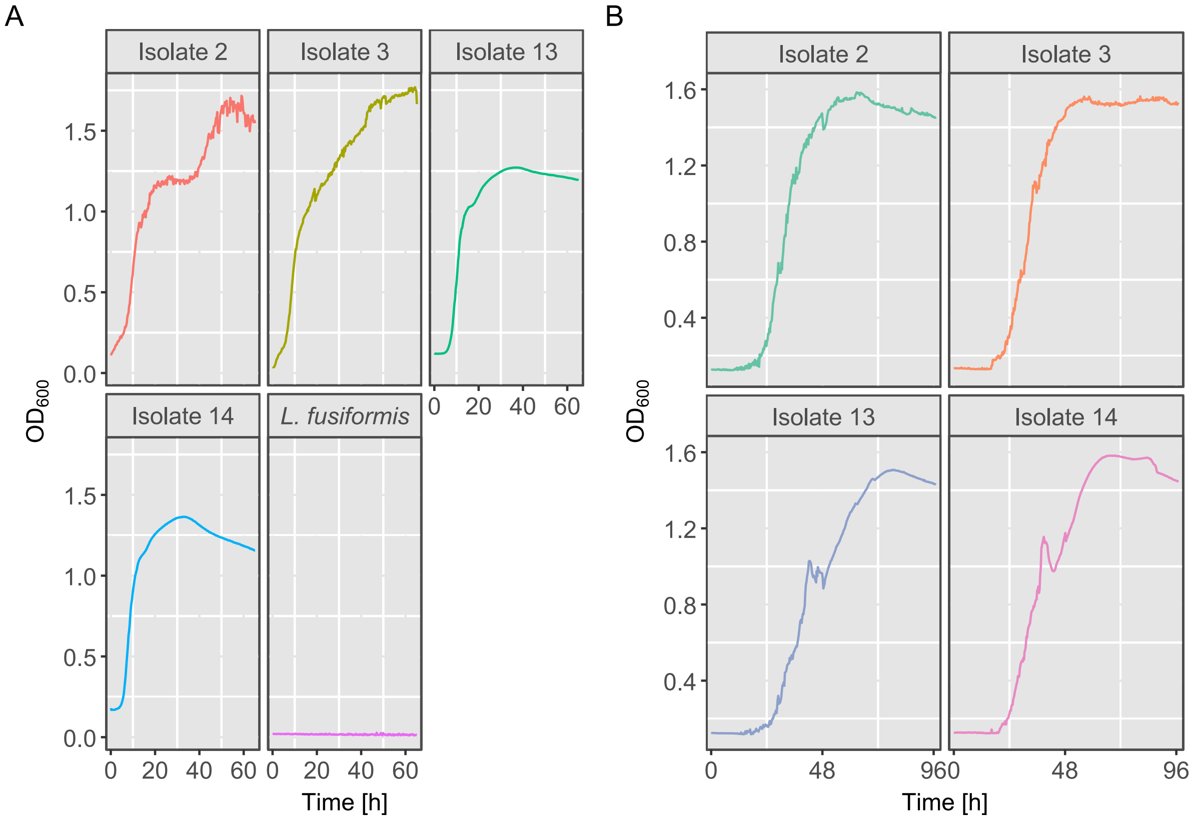

Figure S 4 – OD_{600} growth curves in TSB pH 10 (A) and TSB pH 11(B) for the best growing isolates at TSB pH 10. Isolates were inoculated from a double transferred fully grown suspension in TSB. All curves were inoculated to OD_{600} = 0.01 and are average of three replicates.

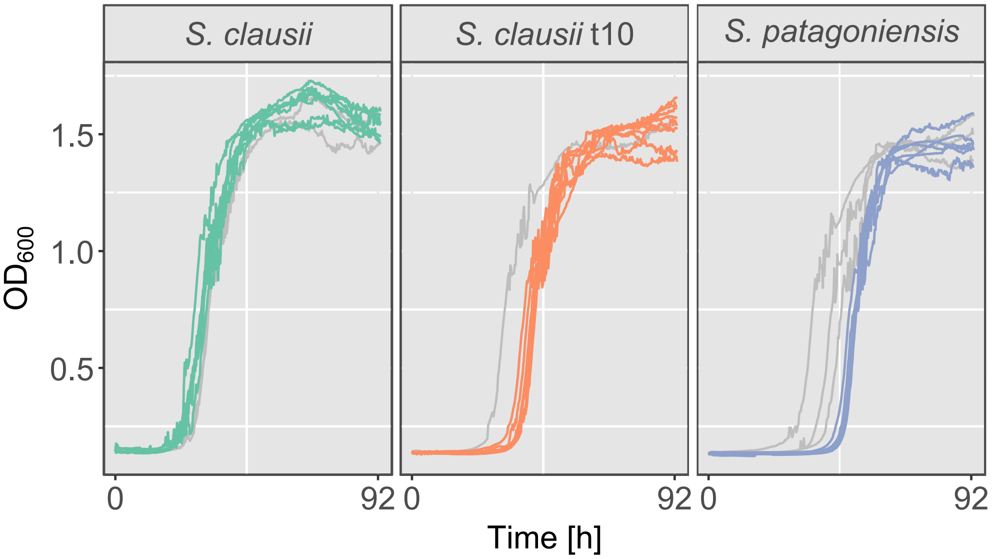

	S. clausii		*S. clausii t10*		*S. patagoniensis*	
	**Average**	**Std**	**Average**	**Std**	**Average**	**Std**
**Max log fitted OD**	0.20	0.01	0.17	0.01	0.16	0.01
**Max log fitted growth rate**	0.17	0.02	0.20	0.01	0.23	0.01
**Lag phase (h)**	20.19	0.59	34.21	0.84	43.69	1.00

Figure S 5 – OD_{600} growth curves in TSB pH 11 for the original isolates and the ALE isolate (S. clausii *t10*). *All curves were inoculated to OD_{600} = 0.01. Isolates were inoculated from a double transferred fully grown suspension in TSB pH 11. Grey outlined curves are outliers based on Iglewicz and Hoaglin's robust test for multiple outliers. The average and standard deviation of the maximum log fitted OD, growth rate and lag phase based on Gompertz fitting for all curves for all three tested isolates is shown. Maximum growth rates showed significant difference between isolates* ($p < 0.05$).

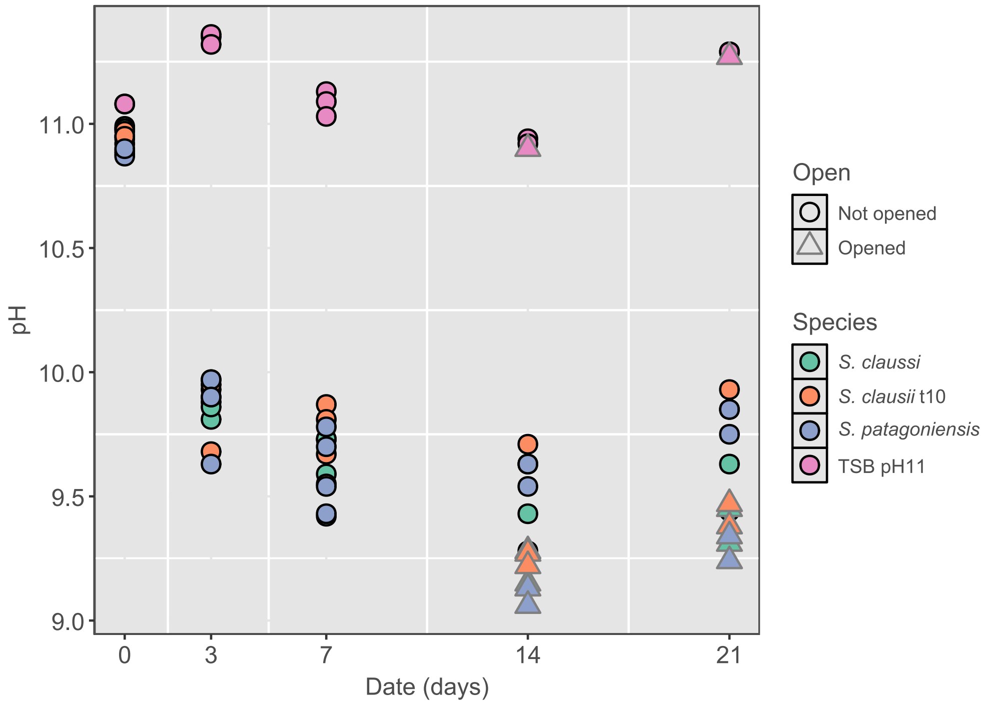

Figure S 6 – pH measured over time for all conditions at day 0, 3, 7, 14 and 21. A steady drop in the pH is observed as CO_2 dissolves in the media until a steady state is achieved. No significant difference was observed between isolates ($p > 0.05$). For the control, TSB pH11 (pink) no significant difference was observed ($p > 0.05$).

Table S 2 – Cell counts in the bacterial suspension over time for the three tested bacteria, S. clausii, the adapted S. clausii and S. patagoniensis. Fully grown bacterial suspensions were used at day 0. A starting population is maintained at least until day 3, but after 7 days in an anoxic environment the population rapidly reduces in size and a small stable population is maintained. Opening the bottles at day 7 leads to an increase in total cell counts for all isolates when measured at 14 days (rows in bold).

Date	Species	Gate	Average	
0	*S. clausii*	Intact	2.0E+05	± 1.1E+05
0	*S. clausii*	Damaged	9.1E+04	± 4.9E+04
0	*S. clausii t10*	Intact	5.4E+05	± 3.6E+04
0	*S. clausii t10*	Damaged	3.1E+05	± 4.0E+04

0	*S. patagoniensis*	Intact	7.4E+05	± 1.7E+05
0	*S. patagoniensis*	Damaged	5.4E+05	± 1.2E+05
3	*S. clausii*	Intact	1.8E+05	± 4.5E+04
3	*S. clausii*	Damaged	5.6E+04	± 2.2E+04
3	*S. clausii t10*	Intact	6.8E+05	± 1.9E+04
3	*S. clausii t10*	Damaged	5.3E+04	± 2.1E+04
3	*S. patagoniensis*	Intact	7.5E+05	± 5.6E+04
3	*S. patagoniensis*	Damaged	7.4E+04	± 4.4E+04
7	*S. clausii*	Intact	1.8E+05	± 3.1E+04
7	*S. clausii*	Damaged	1.1E+05	± 2.4E+04
7	*S. clausii t10*	Intact	6.7E+05	± 5.3E+04
7	*S. clausii t10*	Damaged	1.1E+05	± 1.6E+04
7	*S. patagoniensis*	Intact	5.5E+05	± 2.0E+05
7	*S. patagoniensis*	Damaged	1.7E+05	± 6.3E+04
14	*S. clausii*	Intact	6.1E+04	± 2.7E+04
14	*S. clausii*	Damaged	1.7E+05	± 3.3E+04
**14**	***S. clausii***	**Intact**	**1.5E+05**	± **1.5E+03**
**14**	***S. clausii***	**Damaged**	**9.5E+04**	± **3.6E+04**
14	*S. clausii t10*	Intact	1.8E+05	± 3.4E+04
14	*S. clausii t10*	Damaged	1.3E+05	± 1.1E+04
**14**	***S. clausii t10***	**Intact**	**2.6E+05**	± **6.0E+04**
**14**	***S. clausii t10***	**Damaged**	**2.9E+05**	± **3.1E+02**
14	*S. patagoniensis*	Intact	2.3E+05	± 2.5E+04

14	*S. patagoniensis*	Damaged	2.4E+05	± 7.4E+04
**14**	***S. patagoniensis***	**Intact**	**1.7E+05**	± **4.0E+04**
**14**	***S. patagoniensis***	**Damaged**	**3.9E+05**	± **3.3E+04**

Table S 3 – Detailed calculations for the estimation of CO_2 and dissolved inorganic carbon in the system at day 14. The suspensions tested included a water control (Cont), S. clausii *(Sc),* S. clausii *t10 (Sct10) and* S. patagoniensis *(Sp).*

	**Sample**	**#**	**Opened at day 7**	**[1]CO_2 concentration (%) m[CO2]h**	**[2]Pressure in bottle (kPa)**	**[3]Total pressure (kPa)**	**[3]Total pressure (atm)**	**[3]Partial pressure CO_2 (atm) pCO_2**	**[3]CO_2 concentration in headspace normalized to liquid volume (mol/L)**
						P_T (kPa) = 101.325 + pressure in bottle	P_T (atm) = P_T / 101.3	pCO_2 (g) = $m[CO2]_h$ /100 * P_T	$[CO_2]_h$ (g) = n/V = partial pressure*(V_h/V_l)/(R*T)
1	Cont	1	n	0	-23.0	78.325	0.77	0.0000	0.00E+00
2	Cont	2	n	0	-27.8	73.525	0.73	0.0000	0.00E+00
3	Cont	3	y	0	-13.6	87.725	0.87	0.0000	0.00E+00
4	Cont	4	y	0	-18.4	82.925	0.82	0.0000	0.00E+00
5	*Sc*	1	n	0	-22.4	78.925	0.78	0.0000	0.00E+00
6	*Sc*	2	n	0	-31.7	69.625	0.69	0.0000	0.00E+00
7	*Sc*	3	y	0	-19.4	81.925	0.81	0.0000	0.00E+00
8	*Sc*	4	y	0	-6.3	95.025	0.94	0.0000	0.00E+00
9	*Sct10*	1	n	0	-43.0	58.325	0.58	0.0000	0.00E+00
10	*Sct10*	2	n	0	-43.1	58.225	0.57	0.0000	0.00E+00
11	*Sct10*	3	y	0	-19.2	82.125	0.81	0.0000	0.00E+00
12	*Sct10*	4	y	0.0325	-18.9	82.425	0.81	0.0003	2.14E-05
13	*Sp*	1	n	0.3884	-34.4	66.925	0.66	0.0026	2.08E-04
14	*Sp*	2	n	0.2396	-48.0	53.325	0.53	0.0013	1.02E-04
15	*Sp*	3	y	0.4783	-11.5	89.825	0.89	0.0042	3.43E-04
16	*Sp*	4	y	1.0937	-16.7	84.625	0.84	0.0091	7.39E-04

[1]CO_2 concentration was measured using gas chromatography.
[2]Pressure in the bottle was measured using tensiometer.
[3]Values calculated using the ideal gas law, Henry's law, and the dissolution constants of CO_2.
[4]pH values were measured using a pH probe.

Table S 3 – Detailed calculations for the estimation of CO_2 and dissolved inorganic carbon in the system at day 14 (continued). The suspensions tested included a water control (Cont), S. clausii *(Sc),* S. clausii *t10 (Sct10) and* S. patagoniensis *(Sp).*

	[3]Dissolved CO_2 (mol / Lt)	[3]Concentration H_2CO_3 (mol / Lt)	[4]pH	[3]Concentration of H^+ from pH (mol / Lt)	[3]Concentration HCO_3^- (mol / Lt)	[3]Concentration CO_3^{2-} (mol / Lt)	[3]Total dissolved inorganic carbon (mol / Lt)	[3]Total inorganic carbon (mol / Lt)
	$[CO_2]_{aq}$= pCO_2* 0.034 (mol/L.atm)	$[H_2CO_3]$ = $[CO_2]_{aq}$*1.7x10^{-3}		$[H^+] = 10^{-pH}$	$[HCO_3^-]$= (H_2CO_3/H^+) * 2.5x10^{-4}	$[CO_3^{2-}]$ =(HCO_3^-/H^+)*4.7x10^{-11}	= H_2CO_3 + HCO_3^- + CO_3^{2-} + CO_2 (*l*)	$[C_{inorganics}]$= H_2CO_3 + HCO_3^- + CO_3^{2-}+ CO_2 (*l*) + CO_2 (*g*)
1	0.00E+00	0.00E+00	10.94	1.14815E-11	0.00E+00	0.00E+00	0.00E+00	0.00E+00
2	0.00E+00	0.00E+00	10.92	1.20226E-11	0.00E+00	0.00E+00	0.00E+00	0.00E+00
3	0.00E+00	0.00E+00	10.90	1.25893E-11	0.00E+00	0.00E+00	0.00E+00	0.00E+00
4	0.00E+00	0.00E+00		1	0.00E+00	0.00E+00	0.00E+00	0.00E+00
5	0.00E+00	0.00E+00	9.28	5.24807E-10	0.00E+00	0.00E+00	0.00E+00	0.00E+00
6	0.00E+00	0.00E+00	9.43	3.71535E-10	0.00E+00	0.00E+00	0.00E+00	0.00E+00
7	0.00E+00	0.00E+00	9.28	5.24807E-10	0.00E+00	0.00E+00	0.00E+00	0.00E+00
8	0.00E+00	0.00E+00	9.15	7.07946E-10	0.00E+00	0.00E+00	0.00E+00	0.00E+00
9	0.00E+00	0.00E+00	9.71	1.94984E-10	0.00E+00	0.00E+00	0.00E+00	0.00E+00
10	0.00E+00	0.00E+00	9.63	2.34423E-10	0.00E+00	0.00E+00	0.00E+00	0.00E+00
11	0.00E+00	0.00E+00	9.27	5.37032E-10	0.00E+00	0.00E+00	0.00E+00	0.00E+00
12	8.99E-06	1.53E-08	9.22	6.02560E-10	6.34E-03	4.94E-04	6.84E-03	6.854E-03
13	8.72E-05	1.48E-07	9.54	2.88403E-10	1.29E-01	2.09E-02	1.50E-01	1.496E-01
14	4.29E-05	7.29E-08	9.63	2.34423E-10	7.77E-02	1.56E-02	9.33E-02	9.339E-02

15	1.44E-04	2.45E-07	9.13	7.41310E-10	8.27E-02	5.23E-03	8.80E-02	8.821E-02
16	3.11E-04	5.28E-07	9.06	8.70964E-10	1.52E-01	8.16E-03	1.60E-01	1.604E-01

[1] CO_2 concentration was measured using gas chromatography.
[2] Pressure in the bottle was measured using tensiometer.
[3] Values calculated using the ideal gas law, Henry's law, and the dissolution constants of CO_2.
[4] pH values were measured using a pH probe.

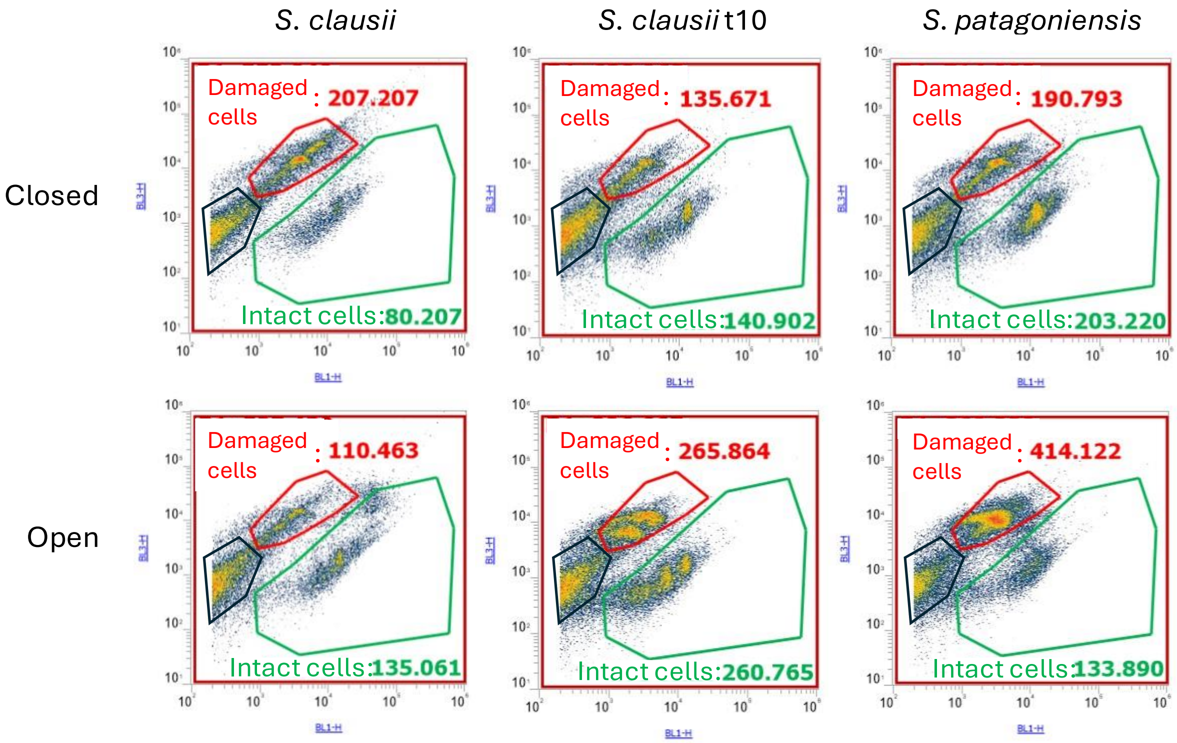

Figure S 7 – Density plots of flow cytometry measurements for all three isolates at day 14. Red gate: damaged cells; green gate: intact cells; blue gate: background. Top plots are from the bottles that remained closed while bottom plots are from the bottles that were opened at day 7. A noticeably denser cloud, for damaged and intact cells, is visible in the opened bottles as compared to the closed ones.

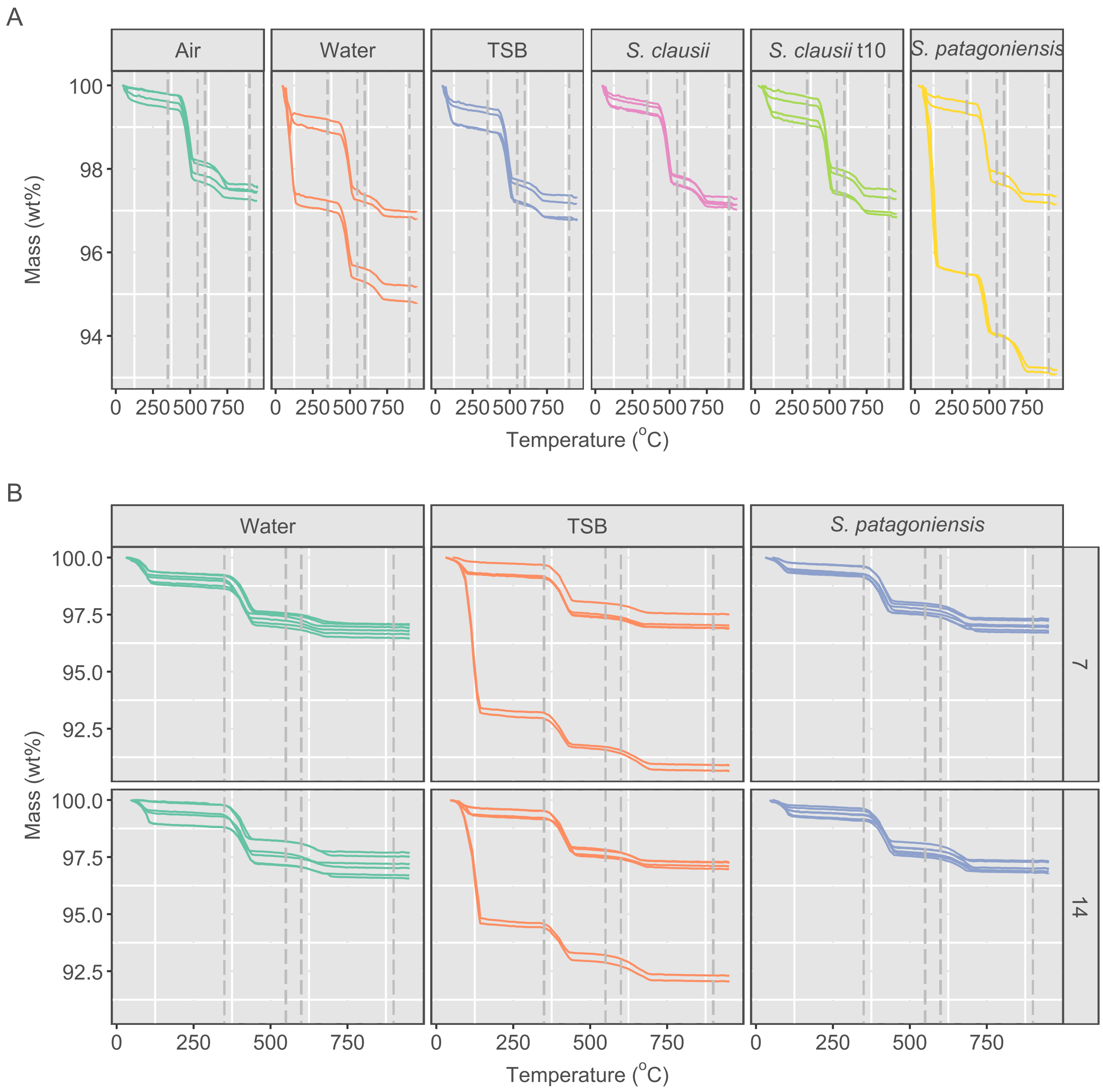

Figure S 8 – A) Thermogravimetry analysis was performed on four different lime mortar samples at 7 days for six different applications. B) Thermogravimetry analysis was performed on six different lime mortar samples at 7 and 14 days for three different applications. Three stages of mass loss are observed: 20 – 100 °C which is the release of free H_2O, 350 – 550 °C which is the release of H_2O from $Ca(OH)_2$ and 650 -950 °C for the release of CO_2 from $CaCO_3$.

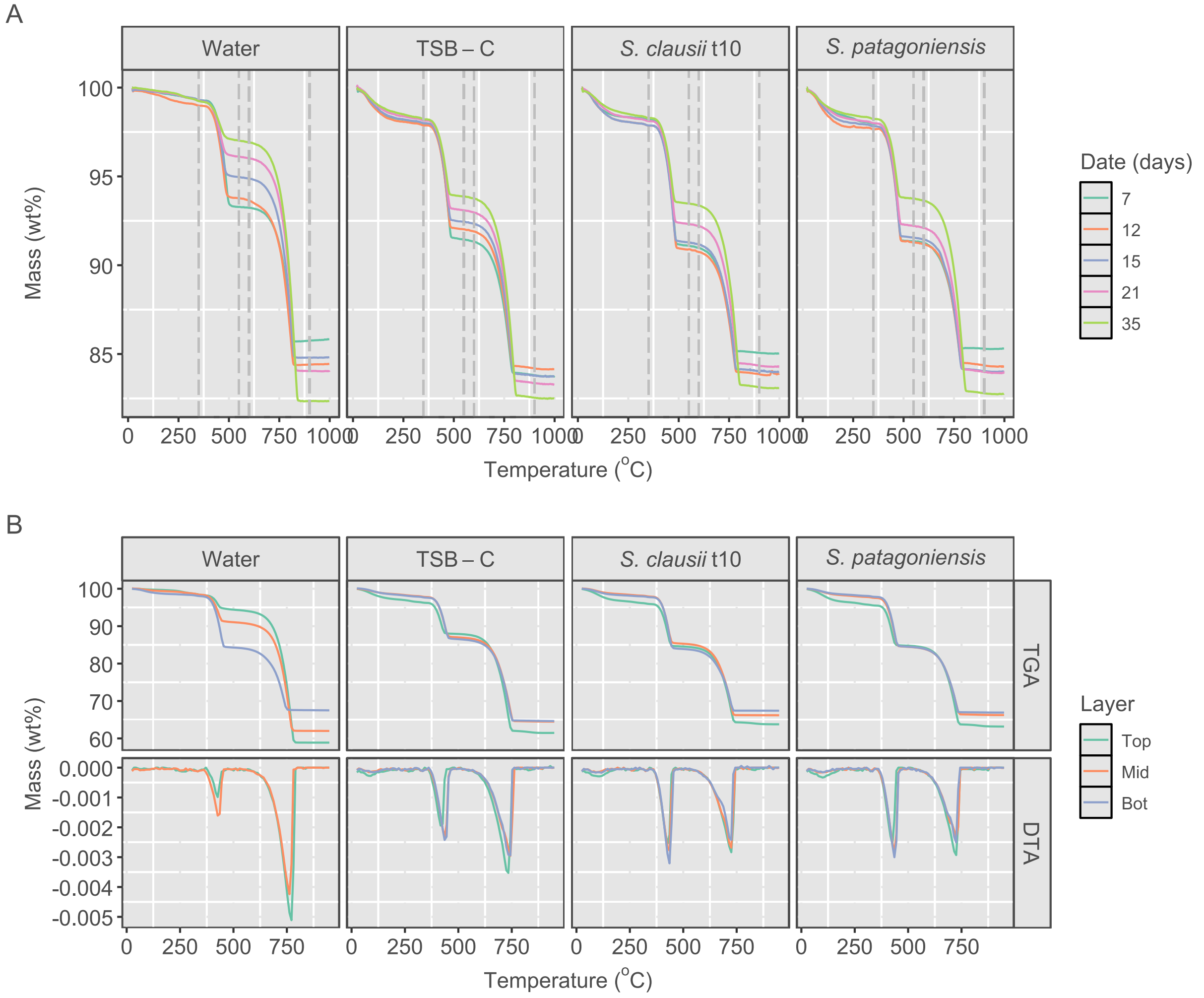

Figure S 9 – Thermogravimetry analysis was performed on lime pastes at 7, 12, 15, 21 and 35 days for four different applications. B) Thermogravimetry analysis was performed on lime pastes samples at 21 days for four different applications. Each sample was grinded into three layers, a top, middle and bottom layer. Three stages of mass loss are observed: 20 – 250 °C which is the release of H_2O and burning of organic matter, 350 – 550 °C which is the release of H_2O from $Ca(OH)_2$ and 650 -950 °C for the release of CO_2 from $CaCO_3$.

89 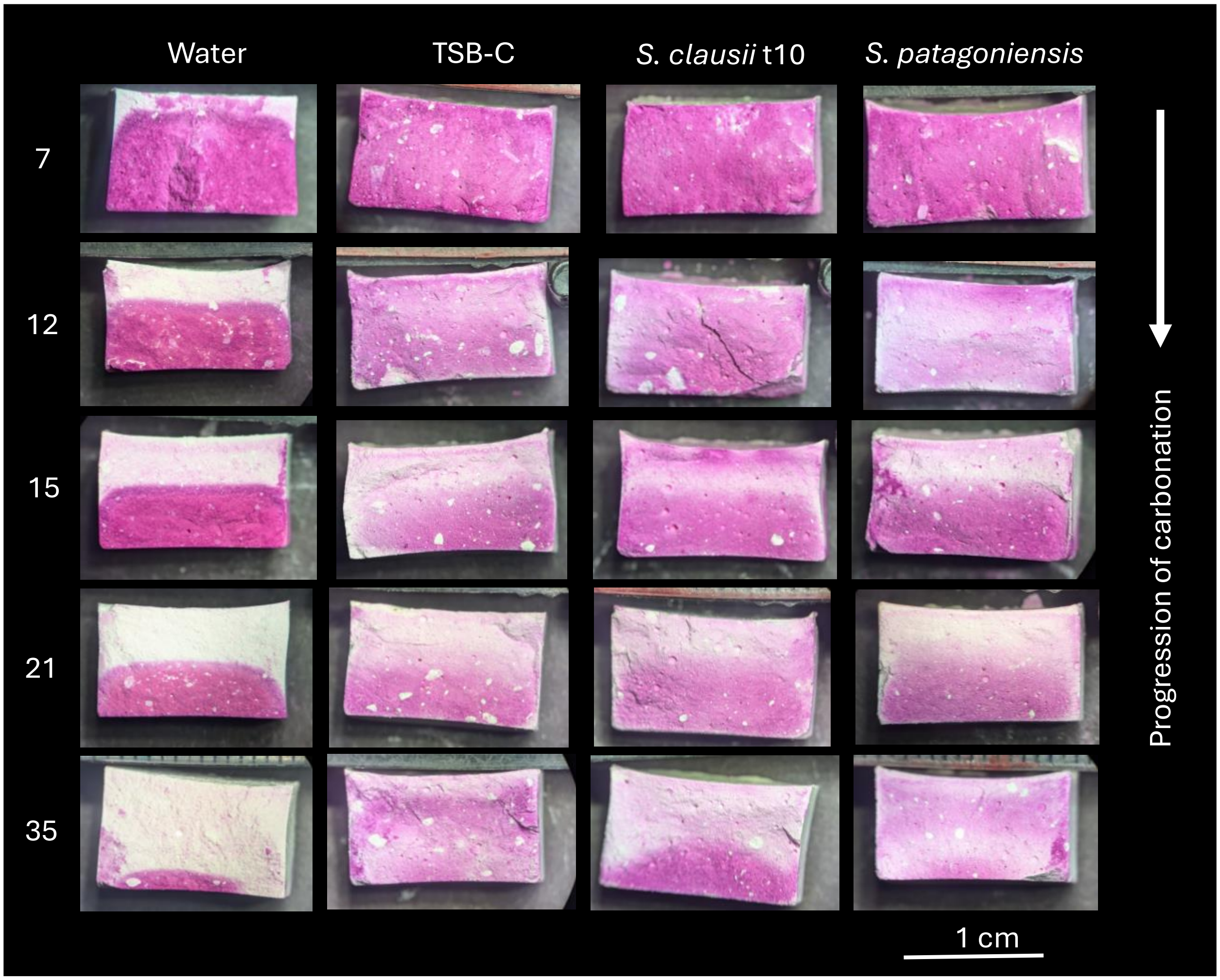

90 *Figure S 10 – Progression of carbonation as measured with spraying of phenolphthalein on a representative sample per*
91 *condition and date. Arrow indicates direction of CO_2 diffusion, and all samples are 1.4 cm wide.*

92
